# Reversed Stability of Zirconium Oxide Dimer Isomers Triggered by Electron Gain or Removal

**DOI:** 10.1021/acs.inorgchem.5c00964

**Published:** 2025-04-03

**Authors:** Dawid Falkowski, Jakub Brzeski, Alicja Mikolajczyk, Piotr Skurski

**Affiliations:** † Faculty of Chemistry, University of Gdańsk, Wita Stwosza 63, 80-308 Gdańsk, Poland; ‡ QSAR Lab Ltd., Trzy Lipy 3, 80-172 Gdańsk, Poland; § Department of Chemistry, University of Utah, Salt Lake City, Utah 84112, United States

## Abstract

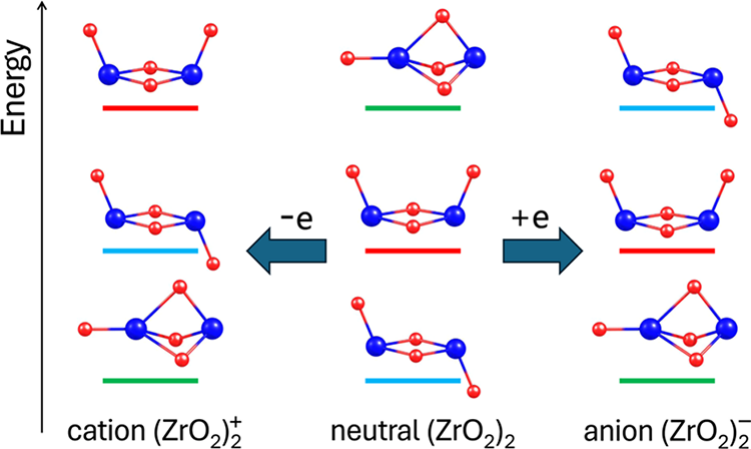

The neutral zirconium oxide dimer and its cationic and anionic counterparts were investigated using advanced ab initio electronic structure methods and flexible basis sets. The exploration of the ground-state potential energy surfaces of (ZrO_2_)_2_, (ZrO_2_)_2_^+^, and (ZrO_2_)_2_^–^ led to the identification of stable isomeric structures and their relative energies. It was found that (ZrO_2_)_2_ adopts three distinct isomeric forms resembling *chair*-, *boat*-, and *scepter*-like structures in its neutral, cationic, and anionic forms. The energetic ordering of these isomers in the neutral dimer is reversed upon either electron attachment or detachment. The adiabatic ionization potential of (ZrO_2_)_2_ was determined to be 9.141 eV, while the adiabatic electron affinity was found to be 1.475 eV. The vertical electron detachment energies were predicted to be 1.949, 1.852, and 1.340 eV, while the vertical ionization potentials, not previously reported in the literature, were determined to be 9.282, 9.375, and 9.594 eV. In both cases, the values correspond to the *scepter*, *boat*, and *chair* isomers, respectively, with the electron detachment energies showing excellent agreement with experimental data. Finally, possible scenarios of isomeric interconversion within the same charge, driven by electron attachment or detachment, were described and analyzed.

## Abstract 2

The neutral zirconium oxide dimer and its cationic and anionic counterparts were investigated using advanced ab initio electronic structure methods and flexible basis sets.

## 1 Introduction

Zirconium dioxide (ZrO_2_), commonly known as zirconia, is one of the most important wide band gap transition metal oxides of exceptional technological and scientific significance. Due to its remarkable combination of chemical, physical, and electronic properties, zirconia has been widely adopted in numerous industrial and research applications, including medicine,^1−8^ advanced ceramics,^9−11^ corrosion-resistant coatings,^12,13^ gas-cleaning technologies,^14^ and heterogeneous catalysis.^15^ In addition, zirconia is a promising candidate for the deep geological disposal of nuclear waste due to its strong radiation-resistant properties.^16−19^

One of the most extensively studied applications of ZrO_2_ is in solid oxide fuel cells (SOFCs), where it serves as an electrolyte material owing to its excellent ionic conductivity, high-temperature stability, and strong chemical resistance.^20−31^ Research in this field has demonstrated that the performance of SOFCs is directly influenced by the properties of zirconia-based electrolytes, motivating further exploration into the fundamental characteristics of ZrO_2_ and its various structural modifications. Furthermore, zirconia has gained attention in solid oxide electrolysis cells (SOECs),^32,33^ where it plays a crucial role in hydrogen production by enabling efficient electrochemical splitting of water at high temperatures. The photosensitization of water on a ZrO_2_ electrode has inspired the development of ZrO_2_-based technologies that exploit its photocatalytic properties for solar-powered hydrogen fuel cells.^34−36^ Despite significant efforts, these technologies are not yet viable, in part, due to the limited mechanistic knowledge required for further development.^37−39^

Zirconia’s role in energy applications extends beyond SOFCs and SOECs. Recent studies have explored its potential use in perovskite solar cells (PSCs), where its high band gap properties influence charge transport and photoconversion efficiency.^40−45^ Unlike titanium dioxide (TiO_2_), which has been traditionally used as an electron-conducting photoelectrode, zirconia has demonstrated promising properties as a nanostructured material that can enhance photovoltaic performance.^42,43^ Investigations into the kinetics of charge transport within PSCs utilizing ZrO_2_ have revealed that electron transfer occurs via localized states within the band gap rather than through conventional conduction band mechanisms.^44−46^

Beyond energy applications, ZrO_2_ plays a critical role in heterogeneous catalysis. Zirconia is frequently employed as a solid catalyst and as a support material for metal nanoparticles, particularly in methanol synthesis catalysts like Cu/ZrO_2_.^47^ The unique interactions between metal centers and zirconia surfaces contribute to strong metal–support interactions (SMSI), enhancing catalytic efficiency in various reactions.^48^ Sulfated zirconia catalysts exhibit strong acidic properties, making them valuable in hydrocarbon conversion processes such as isomerization, alkylation, and cracking.^49^ Furthermore, zirconia-based materials have been utilized in biomass processing, water purification, and greenhouse gas reduction, demonstrating their importance in environmental applications.^50−53^

The structural polymorphism of zirconia further enhances its versatility. At ambient pressure, ZrO_2_ exists in monoclinic, tetragonal, and cubic phases, with phase transitions being controlled by temperature and dopant incorporation.^54^ The addition of yttria (Y_2_O_3_) stabilizes the cubic phase, yielding yttria-stabilized zirconia (YSZ), which has become a cornerstone material in electrochemical applications such as oxygen sensors and high-performance coatings.^32,33,54,55^ Additionally, zirconia, due to its high dielectric constant, has found applications in nanoelectronics, replacing SiO_2_ in transistor gate structures.^56−58^ The growing demand for high-performance dielectric materials in miniaturized electronic devices continues to drive interest in zirconia-based compounds.^59−61^

Despite the extensive research on bulk and thin-film zirconia, relatively little attention has been given to the structures of small zirconium oxide clusters, particularly in terms of their isomerism and charge-dependent stability. Various neutral and anionic bare (ZrO_2_)*_n_* clusters have been extensively studied using PES,^62−64^ Fourier-transform microwave spectroscopy,^65^ matrix-infrared (IR) spectroscopy,^66^ resonant multiphoton ionization,^67^ laser-induced fluorescence,^67^ dispersed fluorescence,^67^ and theoretical methods.^62,66,68−74^ Computational research by Chen et al. determined the crystal growth modes of small (ZrO_2_)_n_ (*n* ≤ 6, 8) clusters.^72^ They identified four structural growth modes—cube-like, ring-like, top-angle-like, and chain-like—based on the most stable structures and electronic energies obtained from DFT calculations, yet these studies did not explore the isomeric diversity of ZrO_2_ oligomers. Gong et al. prepared and characterized dinuclear zirconium oxide clusters, Zr_2_O_2_ and Zr_2_O_4_, using matrix isolation infrared spectroscopy and quantum chemical calculations. Their results indicated that Zr_2_O_2_ clusters were formed through reactions of metal dimers with O_2_ in solid argon upon sample annealing, with theoretical calculations predicting that the Zr_2_O_2_ cluster adopts a planar cyclic structure.^73^ Woodley et al. performed extensive density functional theory calculations on the most stable structures, energies, and vibrational properties of small zirconium oxide clusters (ZrO_2_)*_n_* (*n* = 1–8), but they did not provide detailed insights into the possible isomeric structures of these systems.^69^ More thorough investigations were carried out by von Helden et al., who conducted B3LYP DFT calculations and identified two competitive isomers for neutral (ZrO_2_)_2_, both of which consist of a four-membered ring with the remaining two oxygen atoms either in a *cis* or in a *trans* configuration, having *C*_2*v*_ and *C*_2*h*_ symmetry, respectively. They found that the energies of these two isomers are very comparable, with the *trans* configuration slightly more stable, probably due to reduced electrostatic repulsion between the two free oxygen atoms.^74^ Likely the most comprehensive computational study on small neutral and negatively charged (ZrO_2_)*_n_* (*n* = 2–4) clusters was conducted by Li and Dixon in 2010. They identified the most stable isomers of these systems, predicted the singlet–triplet energy gaps of the neutral species, determined the vertical and adiabatic electron binding energies for the anionic isomers, and simulated the photoelectron spectrum of (ZrO_2_)_2_^–^ anion.^68^

Although numerous studies have investigated the electronic, mechanical, and optical properties of ZrO_2_ oligomers and polymorphs,^75−82^ the isomerism of even a simple system like the ZrO_2_ dimer in its neutral, cationic, and anionic forms remains inadequately addressed. In particular, no research to date has examined the possibility of interconversion between isomers within the same charge (neutral, cationic, or anionic), determined the ionization potentials characterizing the neutral isomers, or mapped their molecular electrostatic potential surfaces. The absence of comprehensive studies on ZrO_2_ dimer isomerism, particularly in its cationic and anionic forms, represents a significant gap in the literature. Specifically, the role of both electron detachment (leading to cationic forms) and excess electron attachment (resulting in anionic forms) in modifying the stability of neutral zirconia clusters remains unresolved. Given this gap, our contribution aims to conduct a detailed quantum chemical analysis of neutral, cationic, and anionic ZrO_2_ dimers. By identifying and characterizing their isomeric structures, relative stabilities, and electronic properties, we seek to elucidate the effects of electron detachment from neutral clusters and excess electron attachment on their structural preferences. The insights gained from this study will contribute to a deeper understanding of zirconia cluster chemistry and may have broader implications for the development of zirconia-based materials in catalysis, energy conversion, and nanoelectronics.

## 2 Methods

The isomeric structures of (ZrO_2_)_2_, (ZrO_2_)_2_^+^, and (ZrO_2_)_2_^–^ and the transition-state structures corresponding to interconversions between isomers within the same charge were determined by applying the second-order Møller–Plesset perturbation method (MP2).^83−85^ The aug-cc-pVTZ basis set^86,87^ was used for oxygen atoms, whereas Stuttgart/Dresden RSC 1997 effective core potential with (8*s*7*p*6*d*)/[6*s*5*p*3*d*] valence basis set (denoted SDD) was utilized for Zr atoms.^88^ In addition, as the excess electron in (ZrO_2_)_2_^–^ is expected to be localized in the vicinity of Zr atoms, the basis set for these atoms was supplemented with additional diffuse and polarization functions. The extra diffuse functions do not share exponent values, and we used even-tempered two-term *s*, two-term *p*, and two-term *d* basis sets. The geometric progression ratio was equal to 2.0, and for each symmetry, we started to build up the exponents of the extra diffuse functions from the lowest exponent of the same symmetry included in the original (8*s*7*p*6*d*)/[6*s*5*p*3*d*] valence basis set designed for Zr (we achieved lowest exponents of 2.7500 × 10^–3^, 5.6308 × 10^–3^, and 7.5000 × 10^–3^ a.u. for the *s*, *p*, and *d* symmetries, respectively). As far as the extra polarization functions are concerned, we used two *f* and one *g* functions (with the exponents of 0.236 (*f*), 0.883 (*f*), and 0.547 (*g*)) centered on Zr atoms, as recommended by Martin and Sundermann.^89^ As a consequence, we obtained the SDD+2*s*2*p*2*d*(diffuse)+2*f*1*g*(polarization) basis set for Zr atoms, which was used in all calculations in this work, as was the aug-cc-pVTZ basis set for O atoms. In each case, we determined the lowest eigenvalue of the atomic orbital overlap matrix to verify that near linear dependency was not an issue.

Our choice of the computational approach described above for studying the electronic structure of ionic and neutral isomers of the zirconia dimer was primarily motivated by the fact that a very similar theoretical approach, namely, the CCSD(T) method combined with an almost identical basis set (albeit smaller, as it lacked two additional diffuse functions of *p* symmetry), was employed by Zheng et al.^62^ and yielded excellent agreement with the experimental value of the adiabatic electron affinity for the neutral ZrO_2_ monomer (1.62 eV vs the experimentally measured 1.64 ± 0.03 eV^63^). Considering that the basis set used in this study is even larger than that employed in previous reliable studies^62^ and that we verified that the addition of an extra set of diffuse *s*, *p*, and *d* functions (leading to SDD+3*s*3*p*3*d*(diffuse)+2*f*1*g*(polarization) basis sets on Zr atoms) results in negligible changes in the calculated excess electron binding energies, we are confident that the obtained values of adiabatic electron affinity and vertical detachment energy are reliable.

For all systems considered, the harmonic vibrational frequencies characterizing the stationary point structure were evaluated at the same level of theory (i.e., MP2/aug-cc-pVTZ(O)/SDD+2*s*2*p*2*d*+2*f*1*g*(Zr)) to ensure that all obtained structures correspond to either true minima or transition states on the potential energy surface. The electronic energies of the systems studied were refined by employing the coupled-cluster method with the single, double, and noniterative triple excitations (CCSD(T)) method^90−93^ and the same basis sets. Both during the geometry optimizations followed by harmonic vibrational frequencies calculations with the MP2 method and while refining the electronic energies using the CCSD(T) method, all electrons in the core and valence shells have been correlated. The intrinsic reaction coordinate (IRC) procedure was employed to confirm the relevance of each reported transition state (see Figure S1 in the Supporting Information).

The relative energies of the isomers were determined with respect to the most stable isomer of the same charge based on the CCSD(T) electronic energies, with the inclusion of zero-point energy corrections (ZPE) obtained using the MP2 method. The aug-cc-pVTZ(O)/SDD+2*s*2*p*2*d*+2*f*1*g*(Zr) basis set was used throughout these calculations.

Since the (ZrO_2_)_2_^+^ and (ZrO_2_)_2_^–^ ions are open-shell systems, we used methods based on an unrestricted Hartree–Fock (UHF) starting point. Hence, it was important to make sure that little (if any) artificial spin contamination enters into the final wave functions. We computed the expectation value ⟨*S*^2^⟩ for the states studied in this work and found values not exceeding 0.753 for doublet ionic species (at the UHF level). Hence, we are confident that spin contamination is not large enough to significantly affect our findings.

The vertical electron detachment energies (VDE) of the anions and the adiabatic electron affinity (EA) of the neutral species were calculated using the supermolecular approach, i.e., by subtracting the energy of the anion from that of the neutral. Similarly, the vertical ionization potentials (VIP) and adiabatic ionization potential (IP) of the neutral molecules were determined by subtracting the energy of the neutral from that of the cation. To ensure consistency in these calculations, the electronic energies employed in the supermolecular approach were obtained at the CCSD(T)/aug-cc-pVTZ(O)/SDD+2*s*2*p*2*d*+2*f*1*g*(Zr) level of theory.

The partial atomic charges were evaluated (using the CCSD electron densities) by the Natural Bond Orbital (NBO) analysis scheme^94−98^ employing the NBO 7.0 software.^99^ All ab initio calculations were performed with the GAUSSIAN16 (Rev.C.01) package.^100^

## 3 Results and Discussion

We present our results in the following order. First, we discuss the characteristics of the neutral system, including its isomeric structures, their relative stability, possible interconversion pathways, and electrostatic potential maps. We then proceed to the analysis of the ionic forms of the (ZrO_2_)_2_ dimer, considering both cationic and anionic species that result from electron detachment or attachment, respectively. Next, we present the calculated vertical and adiabatic ionization potentials of (ZrO_2_)_2_, as well as the vertical electron detachment energies predicted for (ZrO_2_)_2_^–^. Finally, we provide insights into the possible interconversion between isomers of the same charge, occurring after a change in the total number of electrons and the formation of a new charged or neutral species.

### 3.1 Neutral (ZrO_2_)_2_

**Figure 1:**
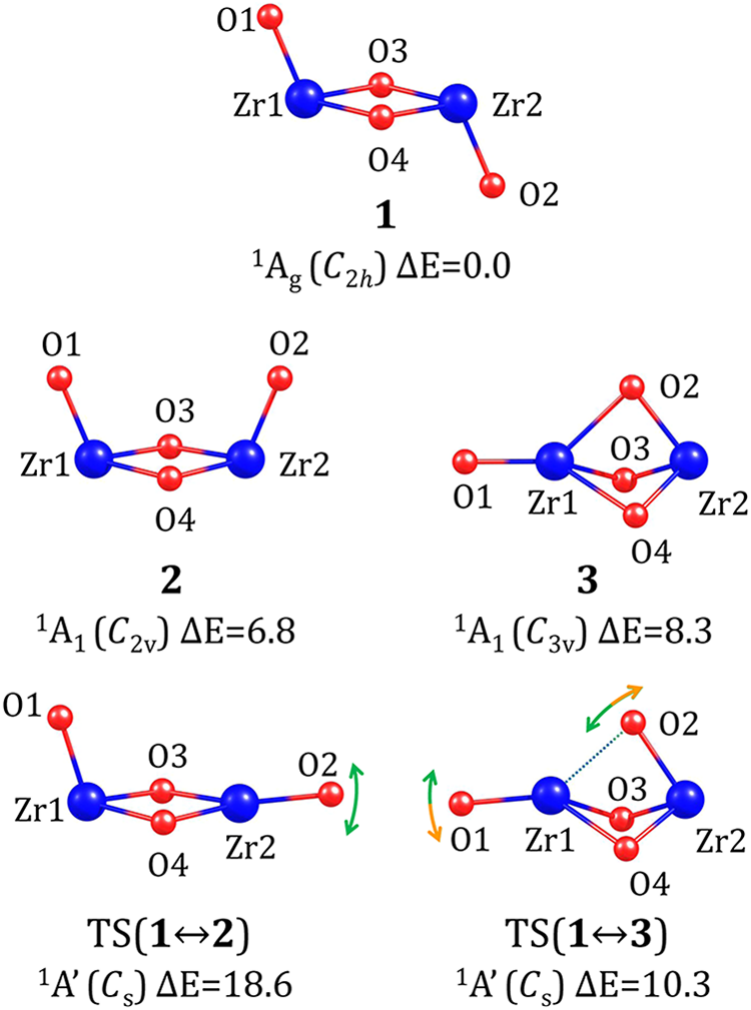
Stationary point structures of neutral (ZrO_2_)_2_ isomers and the transition states corresponding to their interconversions. Relative energies (Δ*E*) are provided in kcal/mol. The symmetry point group is indicated in parentheses. For the transition states (labeled TS), the dotted line represents the bond being formed or ruptured, while the arrows illustrate the approximate directions of the most significant atomic movements along the negative vibrational mode. Relevant structural parameters are summarized in Table 1.

We identified three geometrically stable structures of the neutral zirconium oxide dimer, which we refer to as isomers rather than conformers, primarily because one of these systems differs from the other two in its bonding pattern. Figure 1 presents the structures of these isomers, labeled **1**, **2**, and **3**, in order of increasing energy.

The most stable isomer, **1**, adopts a *C*_2*h*_-symmetry *chair*-like structure, with a *C*_2_ symmetry axis passing through atoms O_3_ and O_4_, a σ*_h_* symmetry plane containing atoms O_1_, Zr_1_, Zr_2_, and O_2_, and an inversion center located at the center of the rhombic fragment formed by atoms Zr_1_, O_3_, Zr_2_, and O_4_. The geometric parameters collected in Table 1 indicate that Zr–O bonds involving oxygen atoms from the rhombic unit (O_3_ and O_4_) are significantly longer (by 0.204 Å) than those involving oxygen atoms connected to a single zirconium atom (i.e., O_1_ and O_2_).

**Table 1:** Structural Parameters of Neutral Isomers of (ZrO_2_)_2_ and the Transition States (TS) Corresponding to Their Interconversions^a^

geometrical parameters	NBO charges
(ZrO_2_)_2_, isomer **1** (*C*_2*h*_) Δ*E* = 0.0	
*r*(Zr_1_–O_1_) = 1.760; *r*(Zr_1_–O_3_) = 1.964; α(O_1_Zr_1_O_3_) = 107.885	*q*(Zr_1,2_) = +2.080
α(Zr_1_O_3_Zr_2_) = 97.288; α(O_1_Zr_1_Zr_2_) = 114.153; γ(Zr_1_O_3_Zr_2_O_4_) = 0.000	*q*(O_1,2_) = −0.951
γ(O_1_Zr_1_Zr_2_O_2_) = 180.000	*q*(O_3,4_) = −1.129
(ZrO_2_)_2_, isomer **2** (*C*_2v_) Δ*E* = 6.8	
*r*(Zr_1_–O_1_) = 1.756; *r*(Zr_1_–O_3_) = 1.971; α(O_1_Zr_1_O_3_) = 108.540	*q*(Zr_1,2_) = +2.063
α(Zr_1_O_3_Zr_2_) = 97.207; α(O_1_Zr_1_Zr_2_) = 112.399; γ(Zr_1_O_3_Zr_2_O_4_) = 4.013	*q*(O_1,2_) = −0.923
γ(O_1_Zr_1_Zr_2_O_2_) = 0.000	*q*(O_3,4_) = −1.140
(ZrO_2_)_2_, isomer **3** (*C*_3v_) Δ*E* = 8.3	
*r*(Zr_1_–O_1_) = 1.767; *r*(Zr_1_–O_2_) = 2.119; *r*(Zr_2_–O_2_) = 1.887	*q*(Zr_1_) = +2.138
α(O_1_Zr_1_O_2_) = 134.993; α(Zr_1_O_2_Zr_2_) = 82.428; γ(Zr_1_O_2_O_3_O_4_) = 63.430	*q*(Zr_2_) = +2.055
	*q*(O_1_) = −0.974
	*q*(O_2,3,4_) = −1.073
(ZrO_2_)_2_, transition state TS(**1** ↔ **2**) (*C*_s_) Δ*E* = 18.6	
*r*(Zr_1_–O_1_) = 1.755; *r*(Zr_1_–O_3_) = 1.984; *r*(Zr_2_–O_3_) = 1.995; *r*(Zr_2_–O_2_) = 1.780	*q*(Zr_1_) = +2.057
α(O_1_Zr_1_O_3_) = 105.465; α(Zr_1_O_3_Zr_2_) = 96.188; α(O_1_Zr_1_Zr_2_) = 107.906	*q*(Zr_2_) = +2.233
α(O_2_Zr_2_Zr_1_) = 173.496; γ(Zr_1_O_3_Zr_2_O_4_) = 4.625; γ(O_1_Zr_1_Zr_2_O_2_) = 0.000	*q*(O_1_) = −0.923
	*q*(O_2_) = −1.015
	*q*(O_3,4_) = −1.176
(ZrO_2_)_2_, transition state TS(**1** ↔ **3**) (*C*_s_) Δ*E* = 10.3	
*r*(Zr_1_–O_1_) = 1.767; *r*(Zr_1_–O_2_) = 2.184; *r*(Zr_2_–O_2_) = 1.867; *r*(Zr_1_–O_3_) = 2.090	*q*(Zr_1_) = +2.137
*r*(Zr_2_–O_3_) = 1.896; α(O_1_Zr_1_O_2_) = 142.819; α(Zr_1_O_2_Zr_2_) = 81.223	*q*(Zr_2_) = +2.053
α(O_1_Zr_1_Zr_2_) = 173.007; α(O_2_Zr_2_O_3_) = 87.355; γ(Zr_1_O_2_O_3_O_4_) = 62.201	*q*(O_1_) = −0.973
	*q*(O_2_) = −1.067
	*q*(O_3,4_) = −1.075

a Bond lengths *r* are given in Å, valence angles α and dihedral angles γ are provided in degrees. NBO partial atomic charges *q* in |e| were calculated with CCSD electron densities. Relative energies (Δ*E*) are given in kcal/mol.

The second most stable structure, labeled **2**, is essentially a conformer of **1**. Its energy is 6.8 kcal/mol higher than that of **1**, and its *boat*-like structure exhibits *C*_2*v*_ symmetry, with a *C*_2_ axis passing through the center of the rhombic framework and perpendicular to it, as well as two σ_v_ symmetry planes—one containing atoms O_1_, Zr_1_, Zr_2_, and O_2_, and the other perpendicular to it, containing atoms O_3_ and O_4_. The Zr–O bond lengths in **2** closely resemble those in **1** (with deviations below 0.01 Å), as does the tilt angle of the O_1_–Zr_1_ and O_2_–Zr_2_ bonds relative to the rhombic unit (with a difference of less than 2° between structures **1** and **2**), see Table 1. The most significant structural difference between **1** and **2** lies in the arrangement of O_1_ and O_2_ atoms, which, in **2**, are positioned on the same side of the rhombic unit plane, whereas in **1**, on opposite sides. This is reflected in the O_1_–Zr_1_–Zr_2_–O_2_ dihedral angle values of 0 and 180°, respectively. It is reasonable to assume that this structural difference destabilizes isomer **2**, due to steric and valence repulsion caused by flagpole interactions between the O_1_ and O_2_ oxygen atoms.

The third locally stable structure of (ZrO_2_)_2_, labeled **3** in Figure 1, exhibits a significantly different atomic arrangement. It contains three oxygen atoms positioned between two zirconium atoms, connected by elongated Zr–O bonds of 2.119 Å, while the fourth oxygen atom is bound to a single zirconium atom via a much shorter bond (1.767 Å), see Table 1. As a result, isomer **3** corresponds to a *scepter*-like structure (or, more precisely, a triangular bipyramid with a vertex extension) and exhibits *C*_3*v*_ symmetry, with a *C*_3_ axis passing through atoms O_1_, Zr_1_, and Zr_2_, along with three vertical symmetry planes, each containing these atoms along with either O_2_, O_3_, or O_4_. The relative energy (Δ*E*) of isomer **3** is 8.3 kcal/mol, which is 1.5 kcal/mol higher than Δ*E* determined for isomer **2**.

Our results for the neutral (ZrO_2_)_2_ system are consistent with those obtained by Li and Dixon,^68^ both in terms of isomeric structures and their relative energies. In particular, the differences in individual bond lengths and bond angles do not exceed 0.06 Å and 4°, respectively, with the theoretical level we applied leading to systematically shorter bonds in all cases. Regarding relative energies, Li and Dixon reported values of 0.0, 6.2, and 6.5 kcal/mol for structures **1**, **2**, and **3**, respectively, predicting the same isomer ordering and an almost identical Δ*E* for **2**, but a noticeably lower relative energy for isomer **3**.

To explore the possibility of interconversion between (ZrO_2_)_2_ isomers, we identified the relevant transition state (TS) structures corresponding to these processes. Since the *chair*-like isomer **1** was confirmed to be the most stable, we considered its possible transformation into either the *boat*-like (**2**) or *scepter*-like (**3**) structure. As a direct, single-step conversion between isomer **2** and **3** (and vice versa) turned out to be impossible (due to significant structural differences), we present in Figure 1 two TS structures, namely those associated with **1** ⇄ **2** and **1** ⇄ **3** interconversions, labeled TS(**1** ↔ **2**) and TS(**1** ↔ **3**), respectively.

**Figure 2:**
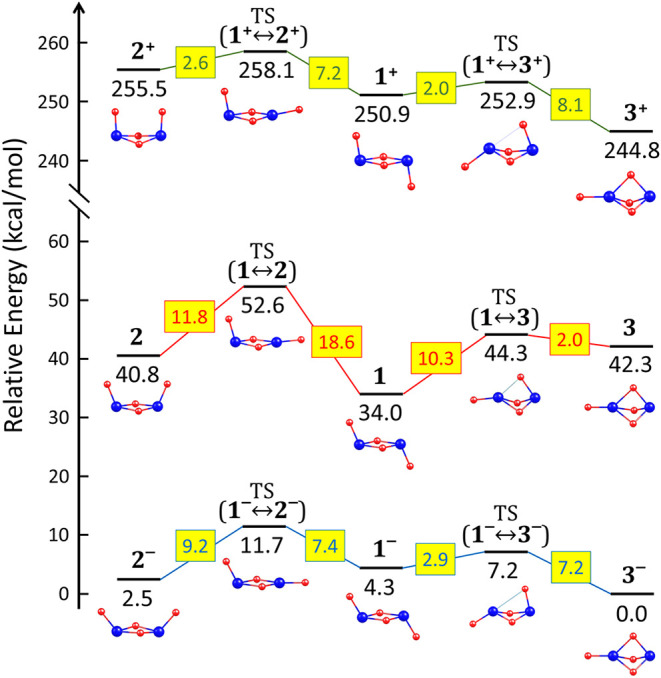
Diagram of relative energies (given in kcal/mol) corresponding to the isomers of the neutral, cationic, and anionic (ZrO_2_)_2_ dimers and the transition states associated with their interconversions. The heights of the corresponding kinetic barriers (also in kcal/mol) are shown in yellow rectangles.

As indicated by the atomic displacements along the negative vibrational mode with a corresponding a’-symmetry imaginary frequency of 165*i* cm^–1^, the transformation between isomers **1** and **2** requires a change in the angle between the Zr_2_–O_2_ bond and the plane of the rhombic unit, see TS(**1** ↔ **2**) in Figure 1 and Table 1. Indeed, in TS(**1** ↔ **2**), this value of 173.5° approximately corresponds to an intermediate geometry between isomers **1** and **2**. The relative energy (with respect to the lowest-energy neutral isomer **1**) was determined to be 18.6 kcal/mol, indicating that the kinetic barrier for the **1** → **2** transformation is 18.6 kcal/mol, whereas for the reverse process (**2** → **1**), this barrier is 11.8 kcal/mol, see Figure 2.

On the other hand, the **1** → **3** transformation requires the simultaneous formation of a new bond between Zr_1_ and O_2_ atoms and a concurrent significant change in the tilt angle of the O_1_–Zr_1_ bond relative to the rhombic unit in isomer **1**. This structural reorganization occurs through atomic displacements along the negative vibrational mode in TS(**1** ↔ **3**), with a corresponding a′-symmetry imaginary frequency of 29*i* cm^–1^ (see Figure 1). Since isomer **3** is substantially higher in energy than isomer **1**, the structure of TS(**1** ↔ **3**) more closely resembles that of **3** rather than **1** (cf. geometrical parameters of **1**, **3**, and TS(**1** ↔ **3**) collected in Table 1). The relative energy of TS(**1** ↔ **3**) was calculated to be 10.3 kcal/mol, meaning that the kinetic barriers for the **1** → **3** and **3** → **1** transformations are 10.3 and 2.0 kcal/mol, respectively, see Figure 2.

An overview of the energy profile including all three neutral isomers (shown in the central part of Figure 2) reveals that the (ZrO_2_)_2_ dimer is expected to exist almost exclusively in the *chair* form (**1**). This is due to its significantly lower energy compared to the *boat* (**2**) and *scepter* (**3**) structures, as well as the fact that any structural evolution of the *chair* isomer requires overcoming substantial (>10 kcal/mol) kinetic barriers. Moreover, as we will demonstrate in the following sections, the low kinetic barrier (2 kcal/mol) for the transformation of isomer **3** into isomer **1** will prove to be significant in the context of (ZrO_2_)_2_ generation from the anion (ZrO_2_)_2_^–^ via excess electron detachment (e.g., during photoelectron spectroscopy measurements).

**Figure 3:**
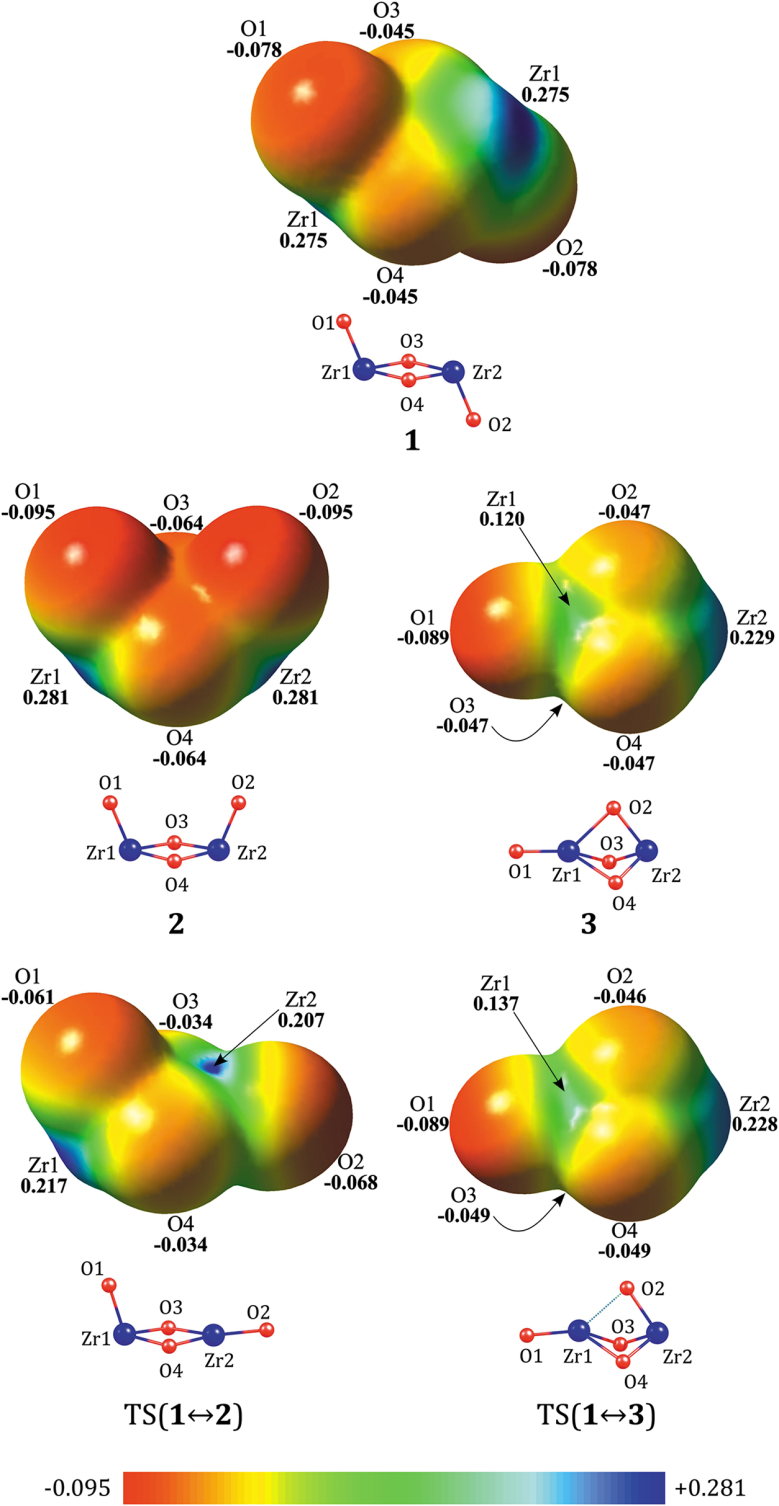
Molecular electrostatic potential maps calculated based on CCSD electron densities and plotted on the 0.001 *e*/bohr^3^ isodensity surface. The values on the electrostatic potential scale are presented in atomic units (a.u.).

Since we aim to investigate not only the neutral (ZrO_2_)_2_ dimer but also its cationic and anionic forms, we calculated the partial atomic charges and molecular electrostatic potential (MEP) maps for all neutral isomers, both based on CCSD electron densities. The NBO population analysis performed for isomers **1**, **2**, and **3** revealed that, as expected, a significant partial positive charge (ca. +2.1|e|) is predicted for Zr atoms, whereas oxygen atoms carry a negative charge. Notably, the oxygen atoms involved in either the rhombic unit (in **1** and **2**) or the triangular bipyramidal fragment (in **3**) exhibit a slightly larger negative charge (ca. −1.1|e|) compared to the remaining oxygen atoms (ca. −0.95|e|), see Table 1. This charge distribution is further supported by the molecular electrostatic potential maps presented in Figure 3, which indicate that the electrostatic potential is positive (ca. 2.8 au for isomers **1** and **2**, and 0.1–0.2 au for isomer **3**) on the chosen isodensity surface in the vicinity of Zr atoms, while negative (ranging from −0.5 to −0.9 au for isomers **1**, **2**, and **3**) in the oxygen atom regions.

Unlike the *chair*-like and *boat*-like isomers, where the Zr atoms are structurally accessible, Zr_1_ in the *scepter*-like isomer appears to be less exposed. As a result, the electrostatic potential value in this region of the isodensity surface is the lowest among the studied structures (0.12 au for isomer **3**). Thus, considering both the positive partial charges predicted by the population analysis for Zr atoms and the positive MEP values in their vicinity, the excess electron in the corresponding anionic systems is expected to be localized in these regions of the molecular framework, similarly to what was demonstrated by Zheng et al. for the monomeric ZrO_2_^–^ anion.^62^

### 3.2 (ZrO_2_)_2_^–^ Anions

Like the monomeric ZrO_2_ molecule,^62^ the isomers of (ZrO_2_)_2_ also form stable anions upon binding an excess electron.^63,68^ On the ground-state doublet anionic potential energy surface (PES), we identified three minima, qualitatively similar to those found for the neutral system. Due to their structural similarity to the neutral forms, we use the same nomenclature (i.e., *chair*, *boat*, and *scepter*) and labels (isomers **1**–**3**). However, we emphasize that, unlike in the case of neutral isomers, these labels do not correspond to the energetic ordering of the anionic species, as reflected in their relative energies. On the contrary, as we are about to demonstrate, this ordering is reversed in the case of anions.

**Figure 4:**
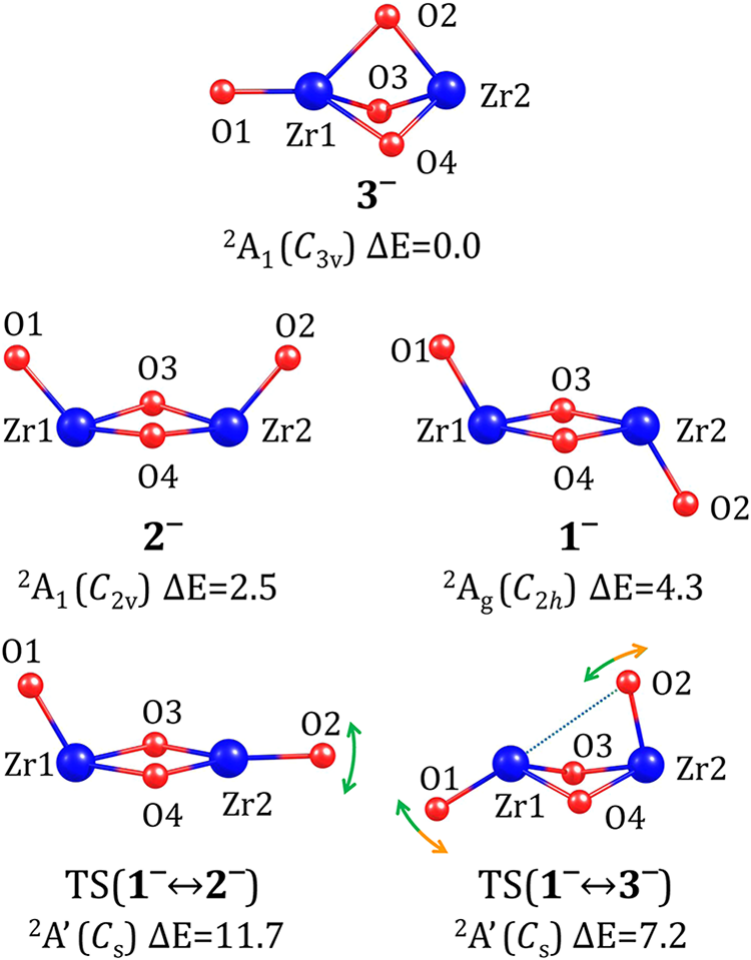
Stationary point structures of anionic (ZrO_2_)_2_^–^ isomers and the transition states corresponding to their interconversions. Relative energies (Δ*E*) are provided in kcal/mol. The symmetry point group is indicated in parentheses. For the transition states (labeled TS), the dotted line represents the bond being formed or ruptured, while the arrows illustrate the approximate directions of the most significant atomic movements along the negative vibrational mode. Relevant structural parameters are summarized in Table 2.

The lowest-energy anionic isomer corresponds to the *C*_3*v*_-symmetry *scepter*-like structure (labeled **3**^**–**^ in Figure 4), which, as described in the preceding section, is the highest-energy isomer on the neutral PES. The bond lengths in **3**^**–**^ differ by less than 0.04 Å compared to those in **3**, while the differences in valence and dihedral angles do not exceed 1.5°, indicating that the structures of the neutral and anionic *scepter* isomers are highly similar (cf. Tables 1 and 2).

**Table 2:** Structural Parameters of Anionic Isomers of (ZrO_2_)_2_ and the Transition States (TS) Corresponding to Their Interconversions^a^

geometrical parameters	NBO charges
(ZrO_2_)_2_^–^, isomer **1**^**–**^ (*C*_2*h*_) Δ*E* = 4.3	
*r*(Zr_1_–O_1_) = 1.785; *r*(Zr_1_–O_3_) = 1.977; α(O_1_Zr_1_O_3_) = 112.222	*q*(Zr_1,2_) = +1.684
α(Zr_1_O_3_Zr_2_) = 96.975; α(O_1_Zr_1_Zr_2_) = 120.339; γ(Zr_1_O_3_Zr_2_O_4_) = 0.000	*q*(O_1,2_) = −1.036
γ(O_1_Zr_1_Zr_2_O_2_) = 180.000	*q*(O_3,4_) = −1.148
(ZrO_2_)_2_^–^, isomer **2**^**–**^ (*C*_2*v*_) Δ*E* = 2.5	
*r*(Zr_1_–O_1_) = 1.784; *r*(Zr_1_–O_3_) = 1.978; α(O_1_Zr_1_O_3_) = 114.251	*q*(Zr_1,2_) = +1.675
α(Zr_1_O_3_Zr_2_) = 95.400; α(O_1_Zr_1_Zr_2_) = 129.606; γ(Zr_1_O_3_Zr_2_O_4_) = 9.067	*q*(O_1,2_) = −1.030
γ(O_1_Zr_1_Zr_2_O_2_) = 0.000	*q*(O_3,4_) = −1.145
(ZrO_2_)_2_^–^, isomer **3**^**–**^ (*C*_3*v*_) Δ*E* = 0.0	
*r*(Zr_1_–O_1_) = 1.806; *r*(Zr_1_–O_2_) = 2.081; *r*(Zr_2_–O_2_) = 1.924	*q*(Zr_1_) = +2.067
α(O_1_Zr_1_O_2_) = 133.530; α(Zr_1_O_2_Zr_2_) = 81.893; γ(Zr_1_O_2_O_3_O_4_) = 62.241	*q*(Zr_2_) = +1.365
	*q*(O_1_) = −1.066
	*q*(O_2,3,4_) = −1.122
(ZrO_2_)_2_^–^, transition state TS(**1**^**–**^ ↔ **2**^**–**^) (*C*_s_) Δ*E* = 11.7	
*r*(Zr_1_–O_1_) = 1.770; *r*(Zr_1_–O_3_) = 2.042; *r*(Zr_2_–O_3_) = 1.958; *r*(Zr_2_–O_2_) = 1.821	*q*(Zr_1_) = +1.370
α(O_1_Zr_1_O_3_) = 110.579; α(Zr_1_O_3_Zr_2_) = 95.383; α(O_1_Zr_1_Zr_2_) = 118.082	*q*(Zr_2_) = +2.125
α(O_2_Zr_2_Zr_1_) = 179.801; γ(Zr_1_O_3_Zr_2_O_4_) = 0.355; γ(O_1_Zr_1_Zr_2_O_2_) = 0.000	*q*(O_1_) = −1.018
	*q*(O_2_) = −1.111
	*q*(O_3,4_) = −1.183
(ZrO_2_)_2_^–^, transition state TS(**1**^**–**^ ↔ **3**^**–**^) (*C_s_*) Δ*E* = 7.2	
*r*(Zr_1_–O_1_) = 1.790; *r*(Zr_1_–O_2_) = 2.962; *r*(Zr_2_–O_2_) = 1.793; *r*(Zr_1_–O_3_) = 1.957	*q*(Zr_1_) = +2.072
*r*(Zr_2_–O_3_) = 1.999; α(O_1_Zr_1_O_2_) = 176.677; α(Zr_1_O_2_Zr_2_) = 66.636	*q*(Zr_2_) = +1.289
α(O_1_Zr_1_Zr_2_) = 147.149; α(O_2_Zr_2_O_3_) = 94.652γ(Zr_1_O_2_O_3_O_4_) = 49.864	*q*(O_1_) = −1.046
	*q*(O_2_) = −1.055
	*q*(O_3,4_) = −1.130

a Bond lengths *r* are given in Å, valence angles α and dihedral angles γ are provided in degrees. NBO partial atomic charges *q* in |e| were calculated with CCSD electron densities. Relative energies (Δ*E*) are given in kcal/mol.

The second lowest-energy isomer of the (ZrO_2_)_2_^–^ anion corresponds to the C_2v_-symmetry *boat*-like structure, labeled **2**^**–**^ (see Figure 4 and Table 2). Although the bond lengths in **2**^**–**^ closely match those in **2** (differences within 0.03 Å), its geometry differs significantly from its neutral counterpart, primarily in the much larger tilt angle (by approximately 17°) of the terminal oxygen atoms relative to the rhomboidal unit (cf. the values of α(O_1_Zr_1_Zr_2_) angles for **2** and **2**^**–**^ in Tables 1 and 2). This structural difference results in the O_1_ and O_2_ atoms in anionic **2**^**–**^ being much farther apart (5.2 Å) than in neutral **2** (4.3 Å). The relative energy of **2**^**–**^ is rather small, amounting to 2.5 kcal/mol (with respect to **3**^**–**^).

In contrast, the highest-energy anionic isomer adopts the *C*_2*h*_-symmetry *chair*-like structure (labeled **1**^**–**^ in Figure 4), which, in the neutral PES, corresponds to the global minimum. In **1**^**–**^, the Zr–O bond lengths remain similar to those in **1**, within 0.03 Å, while some valence angles show moderate deviations. The most pronounced differences are observed in the tilt angles of the terminal O_1_ and O_2_ atoms relative to the rhombic unit, which are 4–6° larger in **1**^**–**^ than in **1** (see Tables 1 and 2). The relative energy of isomer **1**^**–**^ is 4.3 kcal/mol, making it the least stable anionic isomer of (ZrO_2_)_2_^–^.

This analysis reveals that the attachment of an excess electron to (ZrO_2_)_2_ reverses the energetic ordering of its isomers. Specifically, the most stable neutral isomer, namely the *chair*-like system (**1**), becomes the least stable anionic isomer **1**^**–**^, whereas the least stable neutral *scepter*-like isomer (**3**) ends up as the most stable anionic isomer **3**^**–**^. This reordering is a direct consequence of differences in the excess electron binding energies of the **1**^**–**^-**3**^**–**^ isomers, the values of which are provided in the following sections.

Before proceeding with the analysis of possible interconversions between anionic isomers, it is worth noting that the obtained anionic structures and their relative energies are consistent with the results reported by Li and Dixon.^68^ However, the bond lengths determined in our study are systematically slightly shorter (by 0.04–0.06 Å).

As in the case of neutral isomers, no direct (i.e., single-step) conversion between the *scepter* and *boat* anionic isomers is possible. Therefore, only the *scepter* ⇄ *chair* and *boat* ⇄ *chair* interconversions can be considered. The energy profile including all three anionic isomers is presented in Figure 2, showing that the conversion of the most stable isomer **3**^**–**^ into **1**^**–**^ requires overcoming a kinetic barrier of 7.2 kcal/mol, whereas the barrier for the reverse conversion (**1**^**–**^ → **3**^**–**^) is significantly lower (2.9 kcal/mol). The saddle point for these transformations (see structure labeled TS(**1**^**–**^ ↔ **3**^**–**^) in Figure 4) is structurally similar to the corresponding transition state in the neutral system (TS(**1** ↔ **3**) in Figure 1). However, its imaginary frequency is substantially higher (94*i* cm^–1^), and the Zr_1_–O_2_ distance (corresponding to the breaking/forming bond) is longer (cf. Tables 1 and 2). This can be attributed to the greater structural similarity of TS(**1**^**–**^ ↔ **3**^**–**^) to **1**^**–**^ rather than **3**^**–**^, which is a direct consequence of the higher stability of the latter compared to the former. The conversion of isomer **1**^**–**^ into **2**^**–**^ (as well as the reverse transformation) proceeds via the C_s_-symmetry transition state, labeled TS(**1**^**–**^ ↔ **2**^**–**^) in Figure 4. The kinetic barriers for the **1**^**–**^ → **2**^**–**^ and **2**^**–**^ → **1**^**–**^ transitions are similar, amounting to 7.4 and 9.2 kcal/mol, respectively. As a result, the TS(**1**^**–**^ ↔ **2**^**–**^) structure is approximately an intermediate geometry between **1**^**–**^ and **2**^**–**^, which is particularly evident in the tilt angle of the O_2_–Zr_2_ bond relative to the rhomboidal unit. In this saddle point, the corresponding angle is nearly perfectly linear (α(O_2_Zr_2_Zr_1_) = 179.801°, see Table 2). The imaginary vibrational frequency (152*i* cm^–1^) and the remaining geometric parameters determined for TS(**1**^**–**^ ↔ **2**^**–**^) are comparable to those obtained for the corresponding saddle point in the neutral system (see Tables 1 and 2).

**Figure 5:**
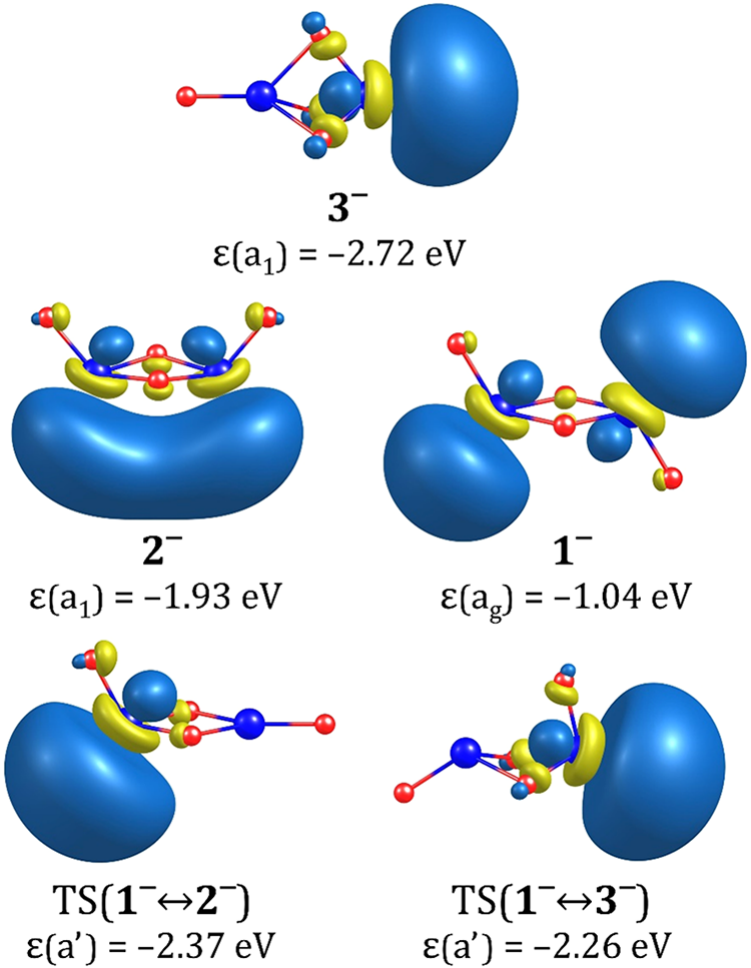
Singly occupied molecular orbitals of isomeric structures of the (ZrO_2_)_2_^–^ anion and the transition states corresponding to their interconversions. The orbital eigenvalues (ε) are provided in eV.

The excess electron density in the **1**^**–**^-**3**^**–**^ anions is primarily localized in the vicinity of both Zr atoms, similarly to the findings of Zheng et al. for the monomeric ZrO_2_^–^ anion.^62^ The singly occupied molecular orbitals (SOMOs) depicted in Figure 5 indicate that, due to symmetry reasons, the fully symmetric SOMOs in **1**^**–**^ and **2**^**–**^ consist of two identical contributions localized near the Zr atoms, away from the O atoms.

In contrast, the singly occupied orbital accommodating the excess electron in the most stable anionic isomer **3**^**–**^ is localized near only one Zr atom, namely Zr_2_, which is the farthest from the oxygen atom forming the vertex extension of the triangular bipyramid.

The eigenvalues of these SOMOs, although providing only a rough approximation of the excess electron binding energy in the respective anions, suggest that the **3**^**–**^ anion is expected to be the most strongly bound (ε(a_1_) = −2.72 eV), whereas the **1**^**–**^ anion appears to be the least strongly bound (ε(a_g_) = −1.04 eV, see Figure 5). As we will demonstrate in the following sections, these rough predictions are consistent with the VDEs determined for the **1**^**–**^, **2**^**–**^, and **3**^**–**^ anions.

### 3.3 (ZrO_2_)_2_^+^ Cations

**Figure 6:**
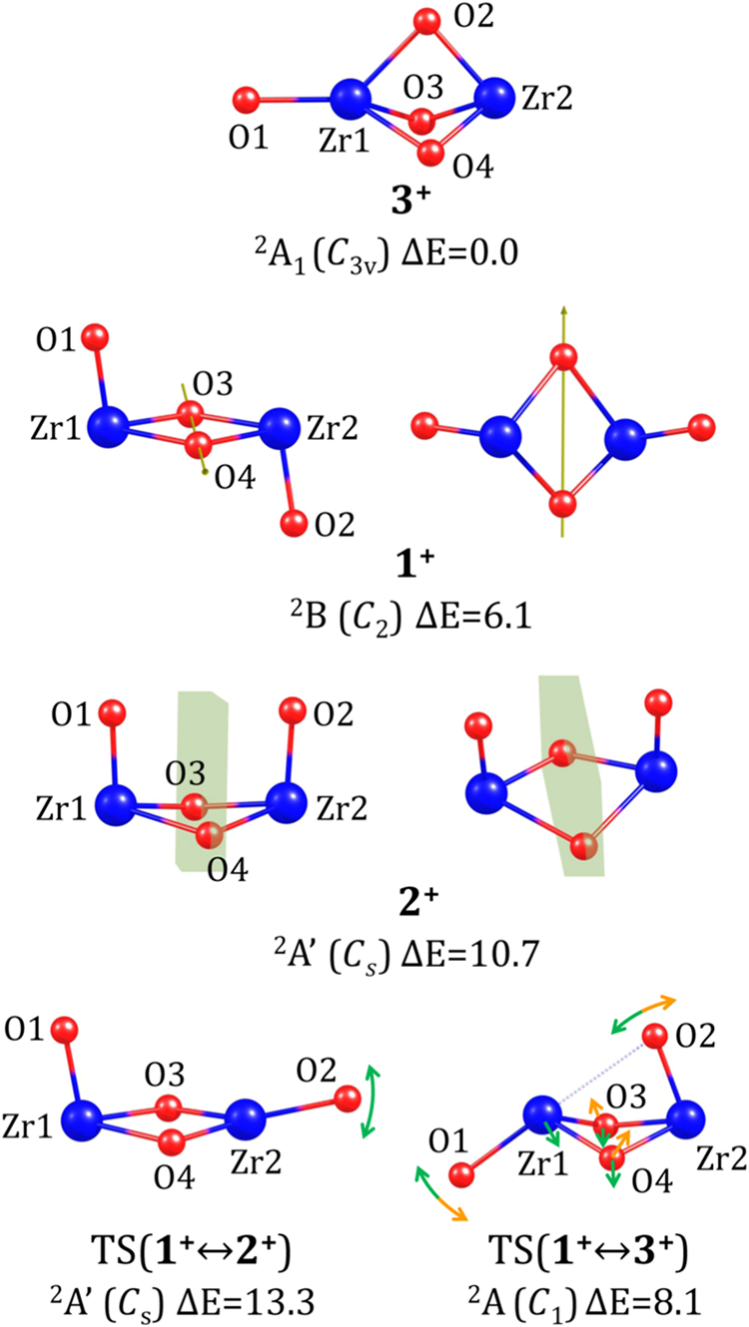
Stationary point structures of cationic (ZrO_2_)_2_^+^ isomers and the transition states corresponding to their interconversions. Relative energies (Δ*E*) are provided in kcal/mol. The symmetry point group is indicated in parentheses. The structures of isomers **1**^**+**^ and **2**^**+**^ are shown from two perspectives, along with their symmetry elements. For the transition states (labeled TS), the dotted line represents the bond being formed or ruptured, while the arrows illustrate the approximate directions of the most significant atomic movements along the negative vibrational mode. Relevant structural parameters are summarized in Table 3.

As in the case of neutral and anionic (ZrO_2_)_2_ systems, we identified three locally geometrically stable isomeric structures for the (ZrO_2_)_2_^+^ cation, which we label using the same convention: *chair* (**1**^**+**^), *boat* (**2**^**+**^), and *scepter* (**3**^**+**^) structures (see Figure 6).

The most stable (ZrO_2_)_2_^+^ system corresponds to the *C*_3*v*_-symmetry *scepter* isomer, whose structure resembles those predicted for the neutral **3** and anionic **3**^**–**^ counterparts. The Zr–O bond lengths in **3**^**+**^ are similar to those in **3** and **3**^**–**^; however, the Zr_1_–O_1_ bond is somewhat longer (by ca. 0.1 Å), while the Zr_1_–O_2,3,4_ bonds are slightly shorter (by ca. 0.1 Å), see Table 3.

**Table 3:** Structural Parameters of Cationic Isomers of (ZrO_2_)_2_ and the Transition States (TS) Corresponding to Their Interconversions^a^

geometrical parameters	NBO charges
(ZrO_2_)_2_^+^, isomer **1**^**+**^ (*C*_2_) Δ*E* = 6.1	
*r*(Zr_1_–O_1_) = 1.723; *r*(Zr_1_–O_3_) = 1.936; *r*(Zr_1_–O_4_) = 2.119	*q*(Zr_1,2_) = +2.152
α(O_1_Zr_1_O_3_) = 101.820; α(O_1_Zr_1_O_4_) = 92.127; α(Zr_1_O_3_Zr_2_) = 105.072	*q*(O_1,2_) = −0.789
α(Zr_1_O_4_Zr_2_) = 92.961; α(O_1_Zr_1_Zr_2_) = 99.536; γ(Zr_1_O_3_Zr_2_O_4_) = 0.000	*q*(O_3_) = −1.114
γ(O_1_Zr_1_Zr_2_O_2_) = 165.956	*q*(O_4_) = −0.612
(ZrO_2_)_2_^+^, isomer **2**^**+**^ (*C*_s_) Δ*E* = 10.7	
*r*(Zr_1_–O_1_) = 1.721; *r*(Zr_1_–O_3_) = 1.942; *r*(Zr_1_–O_4_) = 2.133	*q*(Zr_1,2_) = +2.130
α(O_1_Zr_1_O_3_) = 102.959; α(O_1_Zr_1_O_4_) = 97.147; α(Zr_1_O_3_Zr_2_) = 105.089	*q*(O_1,2_) = −0.763
α(Zr_1_O_4_Zr_2_) = 92.609; α(O_1_Zr_1_Zr_2_) = 92.960; γ(Zr_1_O_3_Zr_2_O_4_) = 17.184	*q*(O_3_) = −1.121
γ(O_1_Zr_1_Zr_2_O_2_) = 0.000	*q*(O_4_) = −0.613
(ZrO_2_)_2_^+^, isomer **3**^**+**^ (*C*_3v_) Δ*E* = 0.0	
*r*(Zr_1_–O_1_) = 1.898; *r*(Zr_1_–O_2_) = 1.992; *r*(Zr_2_–O_2_) = 1.896	*q*(Zr_1_) = +2.258
α(O_1_Zr_1_O_2_) = 133.846; α(Zr_1_O_2_Zr_2_) = 84.570; γ(Zr_1_O_2_O_3_O_4_) = 62.503	*q*(Zr_2_) = +2.286
	*q*(O_1_) = −0.505
	*q*(O_2,3,4_) = −1.013
(ZrO_2_)_2_^+^, transition state TS(**1**^**+**^ ↔ **2**^**+**^) (*C*_s_) Δ*E* = 13.3	
*r*(Zr_1_–O_1_) = 1.719; *r*(Zr_1_–O_3_) = 2.039; *r*(Zr_2_–O_3_) = 1.866; *r*(Zr_2_–O_2_) = 1.884	*q*(Zr_1_) = +2.181
α(O_1_Zr_1_O_3_) = 100.833; α(Zr_1_O_3_Zr_2_) = 98.127; α(O_1_Zr_1_Zr_2_) = 100.479	*q*(Zr_2_) = +2.336
α(O_2_Zr_2_Zr_1_) = 168.473; γ(Zr_1_O_3_Zr_2_O_4_) = 5.867; γ(O_1_Zr_1_Zr_2_O_2_) = 0.000	*q*(O_1_) = −0.811
	*q*(O_2_) = −0.550
	*q*(O_3,4_) = −1.078
(ZrO_2_)_2_^+^, transition state TS(**1**^**+**^ ↔ **3**^**+**^) (*C*_1_) Δ*E* = 8.1	
*r*(Zr_1_–O_1_) = 1.966; *r*(Zr_1_–O_2_) = 2.654; *r*(Zr_2_–O_2_) = 1.754; *r*(Zr_1_–O_3_) = 1.854; *r*(Zr_1_–O_4_) = 1.899; *r*(Zr_2_–O_3_) = 2.021; *r*(Zr_2_–O_4_) = 1.964	*q*(Zr_1_) = +2.283
α(O_1_Zr_1_O_2_) = 177.104; α(Zr_1_O_2_Zr_2_) = 74.177; α(O_1_Zr_1_Zr_2_) = 140.199	*q*(Zr_2_) = +2.209
α(O_2_Zr_1_O_3_) = 67.614; α(O_2_Zr_1_O_4_) = 67.215; α(O_2_Zr_2_O_3_) = 86.521	*q*(O_1_) = −0.511
α(O_2_Zr_2_O_4_) = 88.434; α(Zr_1_O_3_Zr_2_) = 90.471; α(Zr_1_O_4_Zr_2_) = 90.882	*q*(O_2_) = −0.910
α(O_1_Zr_1_O_3_) = 109.491; α(O_1_Zr_1_O_4_) = 112.923; γ(Zr_1_O_2_O_3_O_4_) = 50.743	*q*(O_3_) = −1.022
γ(Zr_2_O_2_O_3_O_4_) = 60.630; γ(Zr_1_O_1_O_3_O_4_) = 47.875	*q*(O_4_) = −1.049

a Bond lengths *r* are given in Å, valence angles α and dihedral angles γ are provided in degrees. NBO partial atomic charges *q* in |e| were calculated with CCSD electron densities. Relative energies (Δ*E*) are given in kcal/mol.

The second lowest-energy isomer of (ZrO_2_)_2_^+^ corresponds to the *chair*-like structure (**1**^**+**^); however, its symmetry (*C*_2_) is lower than that of the corresponding *chair* structures in the neutral and anionic systems (*C*_2*h*_). The absence of a horizontal symmetry plane in **1**^**+**^ results in a deviation of the dihedral angle α(O_1_Zr_1_Zr_2_O_2_) from planarity, shifting by approximately 14° from 180°. This angle describes the tilt of the Zr–O bonds involving the terminal oxygen atoms (O_1_ and O_2_) relative to the plane defined by Zr_1_, O_3_, Zr_2_, and O_4_. These four atoms in **1**^**+**^ do not form a perfect rhombus (as in **1** and **1**^**–**^) but rather a deltoid, with side lengths of 1.936 and 2.119 Å (see Table 3). The relative energy of isomer **1**^**+**^ is 6.1 kcal/mol, which is significantly higher than that of **3**^**+**^, the global minimum on the cationic PES.

The *boat*-like isomer (**2**^**+**^) has the highest relative energy among the cationic species, amounting to 10.7 kcal/mol. Its structure exhibits *C*_*s*_ symmetry (see Figure 6), which is lower than that of the corresponding neutral (**2**) and anionic (**2**^**–**^) structures, both of which possess *C*_2*v*_ symmetry. Unlike in **1**^**+**^, where the Zr_1_, O_3_, Zr_2_, and O_4_ atoms form a planar deltoid, in **2**^**+**^ this unit is distorted due to the nonzero dihedral angle γ(Zr_1_O_3_Zr_2_O_4_) of 17.184°, confirming that the four-atom fragment is not flat (see Table 3). Additionally, the Zr_1,2_–O_3_ and Zr_1,2_–O_4_ bond lengths are unequal, measuring 1.942 and 2.133 Å, respectively. The symmetry plane of **2**^**+**^ (shown in Figure 6) contains the O_3_ and O_4_ atoms, while the Zr–O bonds formed by the terminal oxygen atoms (O_1_ and O_2_) are nearly parallel to it. As a result, the distance between these oxygen atoms (3.261 Å) is significantly shorter than in the neutral (4.295 Å) and anionic (5.200 Å) counterparts.

This analysis reveals that electron withdrawal from neutral (ZrO_2_)_2_ leads to a fundamental reordering of the isomeric energy landscape. Specifically, the *chair* isomer, which constitutes the global minimum on the neutral PES, becomes significantly less stable in its cationic form than the *scepter* isomer, which emerges as the global minimum on the cationic PES. Considering this in a broader context, including the stability of anionic isomers, it can be concluded that a one-electron change in the (ZrO_2_)_2_ system—i.e., ionization via electron detachment or attachment—drastically alters the energetic ordering of the isomers. As a result, the *scepter* structure consistently becomes the most stable ionic isomer, whether cationic or anionic.

The interconversion of cationic isomers via single-step processes is possible only for the *chair* (**1**^**+**^) ⇄ *boat* (**2**^**+**^) and *chair* (**1**^**+**^) ⇄ *scepter* (**3**^**+**^) transformations. In contrast, a direct single-step conversion between the *scepter* (**3**^**+**^) and *boat* (**2**^**+**^) isomers is not feasible due to their substantial structural differences—an observation consistent with the behavior of neutral and anionic isomers, as described in the preceding sections. The structures of the transition states corresponding to the **1**^**+**^ ⇄ **2**^**+**^ and **1**^**+**^ ⇄ **3**^**+**^ interconversions are depicted in Figure 6 (labeled TS(**1**^**+**^ ↔ **2**^**+**^) and TS(**1**^**+**^ ↔ **3**^**+**^), respectively). The energy profile including all three cationic isomers is presented in Figure 2, revealing that the conversion of the most stable **3**^**+**^ isomer into **1**^**+**^ requires overcoming a kinetic barrier of 8.1 kcal/mol, whereas the barrier for the reverse process (**1**^**+**^ → **3**^**+**^) is relatively small (2.0 kcal/mol). The transition state TS(**1**^**+**^ ↔ **3**^**+**^) associated with these transformations has a structure similar to its anionic counterpart but lacks any symmetry elements (except for the identity operation). Even though the atomic displacements along its negative vibrational mode (corresponding to an imaginary vibrational frequency of 97*i* cm^–1^) are primarily dominated by the motion of the O_2_ atom (associated with the breaking/forming of the Zr_1_–O_2_ bond) and the O_1_ atom (involved in the tilt of the O_1_–Zr_1_ bond relative to the Zr_1_–O_3_–Zr_2_–O_4_ fragment), the involvement of O_3_ and O_4_ atoms disrupts any residual symmetry. These atoms shift their positions relative to the plane defined by Zr_1_, O_2_, and Zr_2_, leading to the overall asymmetry of the structure (see the arrows in Figure 6). On the other hand, the kinetic barrier for the **1**^**+**^ → **2**^**+**^ transformation is 7.2 kcal/mol, whereas the barrier for the reverse process is significantly lower, at 2.6 kcal/mol. The TS(**1**^**+**^ ↔ **2**^**+**^) transition state associated with these transformations has a structure similar to that of its anionic counterpart (TS(**1**^**–**^ ↔ **2**^**–**^) on the anionic PES). The atomic displacements along the negative vibrational mode (corresponding to an imaginary frequency of 136*i* cm^–1^) are dominated by the movement of the O_2_ atom within the symmetry plane defined by O_1_, Zr_1_, and Zr_2_.

**Figure 7:**
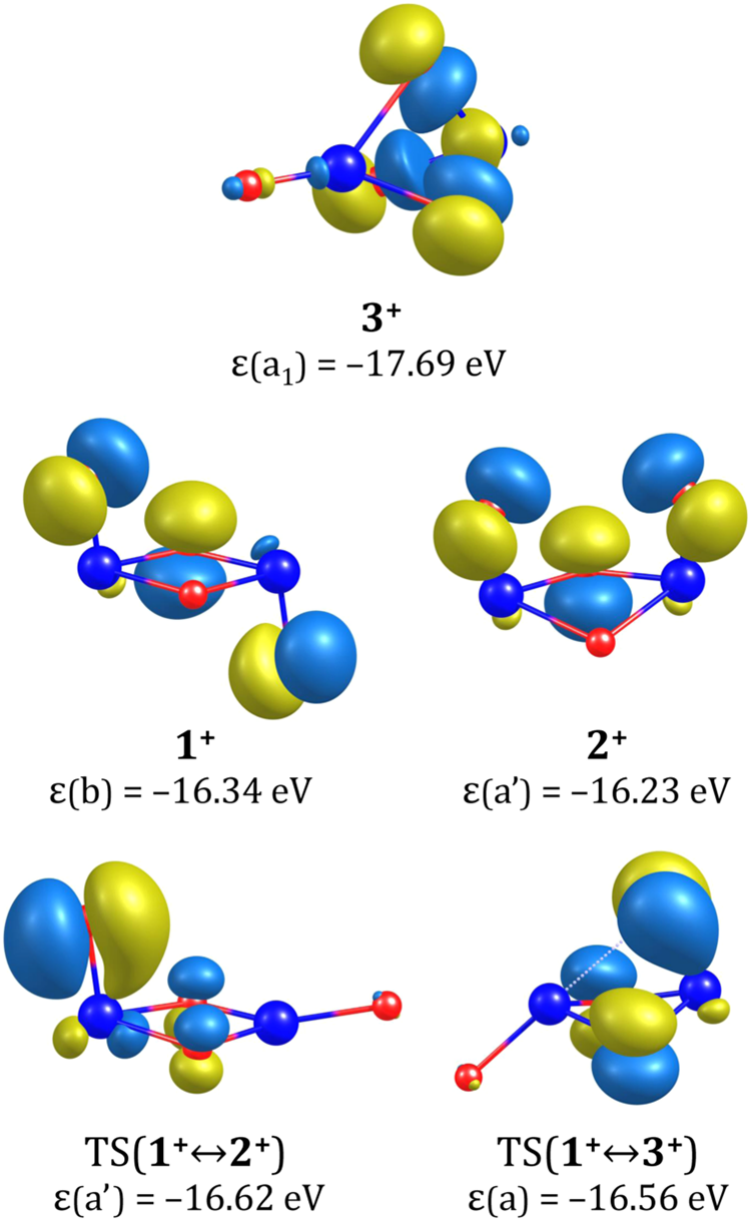
Singly occupied molecular orbitals of isomeric structures of the (ZrO_2_)_2_^+^ cation and the transition states corresponding to their interconversions. The orbital eigenvalues (ε) are provided in eV.

The singly occupied molecular orbitals (SOMOs) for the cationic isomers, shown in Figure 7, provide insight into the orbital that accommodates the unpaired electron in each system. Consequently, they also indicate from which highest doubly occupied molecular orbitals (HOMO) of the neutral system the electron was removed during ionization.

Indeed, these SOMOs correspond to the HOMOs of the respective neutral isomers (not shown here). The most notable differences between the cationic SOMOs and the neutral HOMOs are observed for the *chair* and *boat* isomers, where the symmetry of the cationic forms (**1**^**+**^ (C_2_) and **2**^**+**^ (*C_s_*)) differs from that of their neutral counterparts (**1** (*C*_2*h*_) and **2** (*C*_2*v*_)). Specifically, in **1**^**+**^ and **2**^**+**^, there are no contributions from the atomic orbitals (AOs) of the O_4_ oxygen atom, whereas in **1** and **2**, these contributions are present and symmetrically equivalent to those originating from the AOs of the O_3_ atom. Overall, for all three cationic isomers, the SOMOs are composed of contributions from the 2p atomic orbitals (of the appropriate symmetry) of the oxygen atoms.

### 3.4 Ionization Potentials of Neutral (ZrO_2_)_2_ and Excess Electron Binding Energies of the (ZrO_2_)_2_^–^ Anion

Having discussed the structures and stability of neutral, cationic, and anionic isomers of (ZrO_2_)_2_, as well as the possible interconversions between isomers of the same charge, we now turn to the analysis of transitions between differently charged isomers, which occur due to changes in the total number of electrons in the system. These transitions take place during the measurement of both the ionization potential of neutral species (neutral-to-cation transitions) and the excess electron binding energy of anionic species (anion-to-neutral transitions). We begin this discussion by presenting the adiabatic values, followed by the vertical values determined in our study.

The adiabatic ionization potential of (ZrO_2_)_2_ (labeled IP in Table 4) corresponds to the transition from the singlet ground electronic state of the most stable neutral isomer (*chair* structure, **1**, ^1^A_g_) to the doublet ground state of the most stable cationic isomer (*scepter* structure, **3**^**+**^, ^2^A_1_). We calculated this value to be 9.141 eV, including zero-point energy (ZPE) corrections. Since the IP of neutral (ZrO_2_)_2_ has not yet been reported in the literature, we can only place our predicted value in the context of previously determined adiabatic ionization potentials for the ZrO_2_ monomer (9.4 ± 0.2 eV)^101^ and (ZrO_2_)*_n_* (*n* = 4–6) oligomers (7.6, 7.7, and 8.5 eV for the tetramer, pentamer, and hexamer, respectively).^102^ As this comparison indicates, the adiabatic IP of the zirconium oxide dimer is slightly lower (by ca. 0.3 eV) than that of the monomer, yet significantly higher than those predicted for its larger counterparts.

**Table 4:** Adiabatic Ionization Potential (IP) and Adiabatic Electron Affinity (EA) of (ZrO_2_)_2_ System, Vertical Ionization Potentials (VIP) Characterizing Its Neutral Isomers, and Vertical Electron Detachment Energies (VDE) Characterizing Its Anionic Isomers^a^

system	process	process type	process energy
(ZrO_2_), isomer **1**	**1**, ^1^A_g_ (**1**(*C*_2*h*_)) → **1**^**+**^, ^2^B_g_ (**1**(*C*_2*h*_)) + *e*	vertical	VIP = 9.594 eV
(ZrO_2_), isomer **2**	**2**, ^1^A_1_ (**2**(*C*_2v_)) → **2**^**+**^, ^2^A_1_ (**2**(*C*_2v_)) + *e*	vertical	VIP = 9.375 eV
(ZrO_2_), isomer **3**	**3**, ^1^A_1_ (**3**(*C*_3v_)) → **3**^**+**^, ^2^A_1_ (**3**(*C*_3v_)) + *e*	vertical	VIP = 9.282 eV
(ZrO_2_), isomer **1**	**1**, ^1^A_g_ (**1**(*C*_2*h*_)) → **3**^**+**^, ^2^A_1_ (**3**^**+**^(*C*_3v_)) + *e*	adiabatic	IP = 9.141 eV
(ZrO_2_), isomer **1**	**1**, ^1^A_g_ (**1**(*C*_2*h*_)) + *e* → **3**^**–**^, ^2^A_1_ (**3**^**–**^(*C*_3v_))	adiabatic	EA = 1.475 eV
(ZrO_2_)_2_^–^, isomer **1**^**–**^	**1**^**–**^, ^2^A_g_ (**1**^**–**^(*C*_2*h*_)) → **1**, ^1^A_g_ (**1**^**–**^(*C*_2*h*_)) + *e*	vertical	VDE = 1.340 eV
(ZrO_2_)_2_^–^, isomer **2**^**–**^	**2**^**–**^, ^2^A_1_ (**2**^**–**^(*C*_2v_)) → **2**, ^1^A_1_ (**2**^**–**^(*C*_2v_)) + *e*	vertical	VDE = 1.852 eV
(ZrO_2_)_2_^–^, isomer **3**^**–**^	**3**^**–**^, ^2^A_1_ (**3**^**–**^(*C*_3v_)) → **3**, ^1^A_1_ (**3**^**–**^(*C*_3v_)) + *e*	vertical	VDE = 1.949 eV

a The electron detachment processes corresponding to these values are provided along with information about the involved isomers, their electronic states, and the geometric structure for which the given energy was determined (in parentheses).

The adiabatic electron affinity, labeled EA in Table 4, corresponds to the transition from the singlet ground electronic state of the most stable neutral isomer (*chair* structure, **1**, ^1^A_g_) to the doublet ground state of the most stable anionic isomer (*scepter* structure, **3**^**–**^, ^2^A_1_). Our calculations indicate that this value is 1.475 eV, including ZPE corrections. Although this value is 0.155 eV lower than the adiabatic electron affinity calculated by Li and Dixon (1.63 eV), we believe that their result was slightly overestimated, as were their VDEs for the (ZrO_2_)_2_^–^ isomers, as we are about to demonstrate in the following paragraphs.

The calculated EA value for the zirconium oxide dimer is 0.165 eV lower than the experimentally measured EA of the monomer (1.64 ± 0.03 eV).^62^ This indicates that the formation of the (ZrO_2_)_2_ dimer from two monomers induces a rather small, yet non-negligible, change in the adiabatic electronic stability of the corresponding anions. At the same time, it is worth noting that our calculated EA for the monomer (1.600 eV), obtained using the same computational treatment as for the other results in this study, falls almost entirely within the experimental uncertainty reported by Zheng et al. (±0.03 eV).^62^ This consistency further supports the reliability of the other results presented in this work.

**Figure 8:**
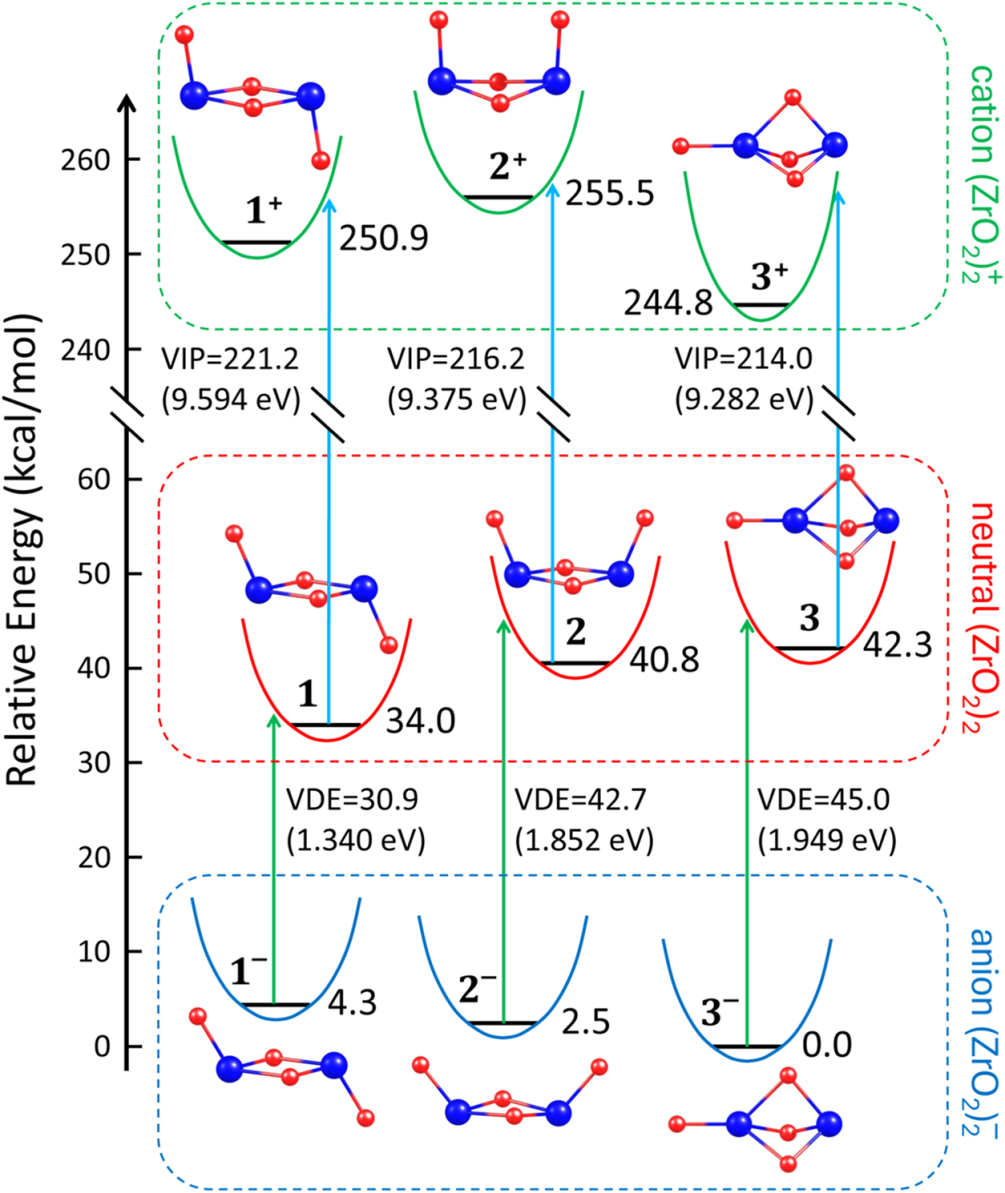
Vertical ionization potentials (VIPs in kcal/mol) of neutral (ZrO_2_)_2_ isomers and vertical electron detachment energies (VDEs in kcal/mol) of isomeric structures of the (ZrO_2_)_2_^–^ anion (the same VIPs and VDEs, expressed in eV, are provided in parentheses). Blue and green arrows represent the vertical process of detaching an electron, leading to either a cationic state or a neutral structure near the local minimum of the corresponding isomer with an additional electron. Relative energies (in kcal/mol), referenced to the energy of the most stable anionic isomer **3**^**–**^ (whose energy is set to zero), are provided for each cationic, neutral, and anionic isomeric structure of (ZrO_2_)_2_.

The vertical ionization potentials (VIPs) and vertical electron detachment energies (VDEs) determined in this study (labeled VIP and VDE in Table 4, respectively) are represented by vertical arrows in Figure 8.

The VIP values predicted for the **1**-**3** neutral isomers span a relatively narrow range of 9.282–9.594 eV, with the highest value (approaching 9.6 eV) corresponding to the most stable neutral isomer **1** (*chair* structure), see Table 4. Although ionization potentials for the zirconium oxide dimer have not yet been reported in the literature, our results suggest that this is the expected value for an experimental measurement capable of determining the vertical ionization potential. Recalling the context of previously reported results for larger ZrO_2_ oligomers, for which VIPs of 7.8, 8.3, and 9.0 eV were predicted for the tetramer, pentamer, and hexamer, respectively,^102^ we can conclude that the VIP values determined for the dimer isomers are higher and more comparable to that of the monomer.

Regarding the vertical electron detachment energies (VDEs) of the anionic **1**^**–**^-**3**^**–**^ isomers, we determined the highest value (1.949 eV) for the most stable **3**^**–**^ isomer, while VDEs of 1.852 and 1.340 eV were calculated for **2**^**–**^ and **1**^**–**^, respectively (see Table 4). A comparison with experimentally measured values reported by Kim et al.^63^ for the *boat* isomer (**2**^**–**^, 1.92 eV) and the *scepter* isomer (**3**^**–**^, 2.00 eV) reveals that our results are in excellent agreement with experimental data, with deviations not exceeding 0.07 eV. Additionally, we note that in both cases, our VDEs are slightly lower than the experimental values^63^ (by 0.05 eV for **3**^**–**^ and 0.07 eV for **2**^**–**^), which should be considered the more accurate reference. Furthermore, it is worth emphasizing that VDEs computed earlier by Li and Dixon^68^ were overestimated by 0.12–0.23 eV relative to the experimental results, making them less reliable.

Finally, we discuss the possible scenarios of isomeric interconversion within the same charge, which are expected to occur in the following cases: (i) electron detachment from the most stable neutral isomer, (ii) electron attachment to the most stable neutral isomer, (iii) electron attachment to the most stable cationic isomer, and (iv) electron detachment from the most stable anionic isomer.

By analyzing these cases step by step, based on the energy profiles presented in Figure 2, we can conclude that

(i) Electron detachment from the most stable neutral isomer (**1**, *chair*) leads to the formation of the **1**^**+**^ cation, which, being less stable than the **3**^**+**^ (*scepter*) isomer, transforms into it by overcoming a small kinetic barrier of 2 kcal/mol.
(ii) Electron attachment to the most stable neutral isomer (**1**, *chair*) results in the formation of the **1**^**–**^ anion, which is expected to readily convert into the most stable anionic isomer (**3**^**–**^, scepter) after surmounting a kinetic barrier of slightly less than 3 kcal/mol.
(iii) Electron attachment to the most stable cationic isomer (**3**^**+**^, *scepter*) generates the neutral **3** isomer, which easily undergoes transformation into the most stable neutral isomer (**1**, *chair*) by overcoming a kinetic barrier of 2 kcal/mol.
(iv) Electron detachment from the most stable anionic isomer (**3**^**–**^, *scepter*) leads to the formation of the neutral **3** isomer, which, being significantly less stable than the neutral **1** (*chair*) isomer, converts into it after overcoming a kinetic barrier of 2 kcal/mol.

The scenarios presented here strongly indicate that, in the case of both neutral and ionic zirconium oxide dimers, only the **1** and **3** isomers (i.e., *chair* and *scepter*) play a significant role. In contrast, the **2** (i.e., *boat*) isomer appears to be largely unimportant in the studied processes, as it is consistently less stable than the other two structures, regardless of whether it is in its cationic, neutral, or anionic form, and is separated from them by substantial kinetic barriers.

## 4 Summary

Based on ab initio electronic structure calculations performed at the CCSD(T) and MP2 levels with the aug-cc-pVTZ/SDD+2*s*2*p*2*d*+2*f*1*g* basis sets, we have analyzed the neutral, cationic, and anionic isomers of the zirconium oxide dimer and arrived at the following conclusions:

(i) The (ZrO_2_)_2_ dimer adopts three isomeric forms resembling *chair*-, *boat*-, and *scepter*-like structures in its neutral, monocationic, and monoanionic forms. The *scepter* isomers exhibit C_3*v*_ symmetry, irrespective of charge. In contrast, the *chair* isomers exhibit *C*_2*h*_ symmetry for the neutral and anionic forms but *C*_2_ symmetry for the cationic form, while the *boat* isomers adopt *C*_2*v*_ symmetry for the neutral and anionic species and *C*_*s*_ symmetry for the cationic species.
(ii) For the neutral dimer, the *chair* isomer is the most stable, with the *boat* and *scepter* structures lying 6.8 and 8.3 kcal/mol higher in energy, respectively.
(iii) The attachment of an excess electron to the neutral dimer reverses the energetic order of the isomers, as the *scepter* anion becomes the most stable form, while the relative energies of the anionic *boat* and *chair* structures are 2.5 and 4.3 kcal/mol, respectively.
(iv) Electron detachment from the neutral dimer also alters the isomeric stability hierarchy, with the *scepter* cation emerging as the most stable species, whereas the cationic *chair* and *boat* isomers lie 6.1 and 10.7 kcal/mol higher in energy, respectively.
(v) The highest stability of the *scepter* structures (characterized by the smallest volume and the highest number of Zr–O bonds) for the ionic forms of the zirconium oxide dimer may indicate that the use of dopants with strong electron-accepting properties or facile electron donation should stabilize the cubic zirconia crystalline phase, which is characterized by the highest packing density.
(vi) The adiabatic ionization potential (IP) of (ZrO_2_)_2_ was determined to be 9.141 eV, while the adiabatic electron affinity (EA) was found to be 1.475 eV.
(vii) The vertical detachment energies (VDEs) of the anionic *scepter*, *boat*, and *chair* isomers were calculated to be 1.949, 1.852, and 1.340 eV, respectively. For the *scepter* and *boat* isomers, these values are in excellent agreement with the available experimental data (no experimental value is reported for the *chair* anion).
(viii) The reversal of the energetic order of isomers upon excess electron attachment results from differences in their vertical detachment energies (VDE(**3**^**–**^) > VDE(**2**^**–**^) > VDE(**1**^**–**^)). The resulting energy lowering is sufficient to overcome the small energy differences between the neutral isomers, making the least stable neutral structure (*scepter*) the most stable anionic form.
(ix) The vertical ionization potentials (VIPs) of the neutral *scepter*, *boat*, and *chair* isomers, which have not been previously reported in the literature, were determined to be 9.282, 9.375, and 9.594 eV, respectively.
(x) Following electron attachment or detachment processes, which involve the most stable isomer of each charge state as the initial species, the system is predicted to quickly relax to the most stable isomer of the corresponding final charge state (i.e., *chair* for the neutral dimer, *scepter* for both the cation and anion). This is facilitated by low kinetic barriers (2–3 kcal/mol) associated with the *chair*(cation) → *scepter*(cation), *chair*(anion) → *scepter*(anion), and *scepter*(neutral) → *chair*(neutral) transformations.

## Supporting Information Available

Cartesian coordinates of all systems and IRC energy profiles (PDF)

## References

[ref1] Patil N. A.; Kandasubramanian B. Biological and mechanical enhancement of zirconium dioxide for medical applications. Ceram. Int. 2020, 46, 4041–4057. 10.1016/j.ceramint.2019.10.220.

[ref2] Chen T.-W.; Vasantha A. S.; Chen S.-M.; Al Farraj D. A.; Elshikh M. S.; Alkufeidy R. M.; Al Khulaifi M. M. Sonochemical synthesis and fabrication of honeycomb like zirconium dioxide with chitosan modified electrode for sensitive electrochemical determination of anti-tuberculosis (TB) drug. Ultrason. Sonochem. 2019, 59, 104718 10.1016/j.ultsonch.2019.104718. 31442770

[ref3] Hingsammer L.; Grillenberger M.; Schagerl M.; Malek M.; Hunger S. Biomechanical testing of zirconium dioxide osteosynthesis system for Le Fort I advancement osteotomy fixation. J. Mech. Behav. Biomed. Mater. 2018, 77, 34–39. 10.1016/j.jmbbm.2017.09.004. 28888931

[ref4] Ahmed S.; Zhang M.; Koval V.; Zou L.; Shen Z.; Chen R.; Yang B.; Yan H. Terahertz probing of low-temperature degradation in zirconia bioceramics. J. Am. Ceram. Soc. 2022, 105, 1106–1115. 10.1111/jace.18139.

[ref5] Nakonieczny D. S.; Ziębowicz A.; Paszenda Z. K.; Krawczyk C. Trends and perspectives in modification of zirconium oxide for a dental prosthetic applications – A review. Biocybern. Biomed. Eng. 2017, 37, 229–245. 10.1016/j.bbe.2016.10.005.

[ref6] Nezhad E. Z.; Sarraf M.; Musharavati F.; Jaber F.; Wang J. I.; Hosseini H. R. M.; Bae S.; Chowdhury M.; So H.; Sukiman N. L. Effect of zirconia nanotube coating on the hydrophilicity and mechanochemical behavior of zirconium for biomedical applications. Surf. Interfaces 2022, 28, 101623 10.1016/j.surfin.2021.101623.

[ref7] Lei Y.; Bian H.; Fu W.; Song X.; Feng J.; Long W.; Niu H. Evaluation of Biomedical Ti/ZrO_2_ Joint Brazed with Pure Au Filler: Microstructure and Mechanical Properties. Metals 2020, 10, 526 10.3390/met10040526.

[ref8] Isacfranklin M.; Dawoud T.; Ameen F.; Ravi G.; Yuvakkumar R.; Kumar P.; Hong S. I.; Velauthapillai D.; Saravanakumar B. Synthesis of highly active biocompatible ZrO_2_ nanorods using a bioextract. Ceram. Int. 2020, 46, 25915–25920. 10.1016/j.ceramint.2020.07.076.

[ref9] Dudczig S.; Veres D.; Aneziris C. G.; Skiera E.; Steinbrech R. W. Nano- and micrometre additions of SiO_2_, ZrO_2_ and TiO_2_ in fine grained alumina refractory ceramics for improved thermal shock performance. Ceram. Int. 2012, 38, 2011–2019. 10.1016/j.ceramint.2011.10.036.

[ref10] Aneziris C. G.; Dudczig S.; Gerlach N.; Berek H.; Veres D. Thermal shock performance of fine grained Al_2_O_3_ ceramics with TiO_2_ and ZrO_2_ additions for refractory applications. Adv. Eng. Mater. 2010, 12, 478–485. 10.1002/adem.201000037.

[ref11] Chen M.; Lu C.; Yu J. Improvement in performance of MgO–CaO refractories by addition of nano-sized ZrO_2_. J. Eur. Ceram. Soc. 2007, 27, 4633–4638. 10.1016/j.jeurceramsoc.2007.04.001.

[ref12] Henrich V. E.; Cox P. A. The Surface Science of Metal Oxides; Cambridge University Press, 1996.

[ref13] Shishkovsky I.; Yadroitsev I.; Bertrand P.; Smurov I. Alumina–zirconium ceramics synthesis by selective laser sintering/melting. Appl. Surf. Sci. 2007, 254, 966–970. 10.1016/j.apsusc.2007.09.001.

[ref14] Sjoholm P.; Ingham D. B.; Lehtimaki M.; Perttu-Roiha L.; Goodfellow H.; Torvela H. Gas-cleaning technology. In Industrial Ventilation Design Guidebook; Elsevier, 2001; pp 1197–1316.

[ref15] Gogotsi G. A. Deformational behaviour of ceramics. J. Eur. Ceram. Soc. 1991, 7, 87–92. 10.1016/0955-2219(91)90005-K.

[ref16] Meldrum A.; Boatner L. A.; Ewing R. C. Nanocrystalline Zirconia Can Be Amorphized by Ion Irradiation. Phys. Rev. Lett. 2001, 88, 025503 10.1103/PhysRevLett.88.025503. 11801024

[ref17] Trachenko K. Understanding resistance to amorphization by radiation damage. J. Phys.: Condens. Matter 2004, 16, R1491–R1515. 10.1088/0953-8984/16/49/R03.

[ref18] Degueldre C. Zirconia inert matrix for plutonium utilisation and minor actinides disposition in reactors. J. Alloys Compd. 2007, 444–445, 36–41. 10.1016/j.jallcom.2006.11.203.

[ref19] Lumpkin G. R. Physical and chemical characteristics of baddeleyite (monoclinic zirconia) in natural environments: an overview and case study. J. Nucl. Mater. 1999, 274, 206–217. 10.1016/S0022-3115(99)00066-5.

[ref20] Liu K. Y.; Fan L.; Yu C. C.; Su P. C. Thermal stability and performance enhancement of nano-porous platinum cathode in solid oxide fuel cells by nanoscale ZrO_2_ capping. Electrochem. Commun. 2015, 56, 65–69. 10.1016/j.elecom.2015.04.008.

[ref21] Oh J.; Seo G.; Kim J.; Bae S.; Park J. W.; Hwang J. H. Plasma-enhanced atomic layer deposition of zirconium oxide thin films and its application to solid oxide fuel cells. Coatings 2021, 11, 362 10.3390/coatings11030362.

[ref22] Saeidpour F.; Zandrahimi M.; Ebrahimifar H. Pulse Electrodeposition of Cobalt/Zirconia Coatings: Oxidation and Electrical Performance of Ferritic Stainless Steel Interconnects. Oxid. Met. 2020, 93, 87–104. 10.1007/s11085-019-09948-4.

[ref23] Cho H. J.; Choi G. M. Effect of milling methods on performance of Ni-Y_2_O_3_-stabilized ZrO_2_ anode for solid oxide fuel cel. J. Power Sources 2008, 176, 96–101. 10.1016/j.jpowsour.2007.09.118.

[ref24] Suzuki T.; Kosacki I.; Anderson H. U. Microstructure–electrical conductivity relationships in nanocrystalline ceria thin films. Solid State Ionics 2002, 151, 111–121. 10.1016/S0167-2738(02)00589-1.

[ref25] Kosacki I.; Rouleau C. M.; Becher P. J.; Bentley J.; Lowndes D. H. Nanoscale effects on the ionic conductivity in highly textured YSZ thin films. Solid State Ionics 2005, 176, 1319–1326. 10.1016/j.ssi.2005.02.021.

[ref26] Shim J. H.; Chao C.-C.; Huang H.; Printz F. B. Atomic Layer Deposition of Yttria-Stabilized Zirconia for Solid Oxide Fuel Cells. Chem. Mater. 2007, 19, 3850–3854. 10.1021/cm070913t.

[ref27] Maier J. Nanoionics: ion transport and electrochemical storage in confined systems. Nat. Mater. 2005, 4, 805–815. 10.1038/nmat1513. 16379070

[ref28] Sata N.; Eberman K.; Eberl K.; Maier J. Mesoscopic fast ion conduction in nanometre-scale planar heterostructures. Nature 2000, 408, 946–949. 10.1038/35050047. 11140675

[ref29] Guo X. X.; Matei I.; Lee J.-S.; Maier J. Ion conduction across nanosized CaF_2_/BaF_2_ multilayer heterostructures. Appl. Phys. Lett. 2007, 91, 103102 10.1063/1.2779254.

[ref30] Garcia-Barriocanal J. G.; Rivera-Calzada A.; Varela M.; Sefrioui Z.; Iborra E.; Leon C.; Pennycook S. J.; Santamaria J. Colossal ionic conductivity at interfaces of epitaxial ZrO_2_: Y_2_O_3_/SrTiO_3_ heterostructures. Science 2008, 321, 676–680. 10.1126/science.1156393. 18669859

[ref31] Ramanathan S. Interface-mediated ultrafast carrier conduction in oxide thin films and superlattices for energy. J. Vac. Sci. Technol. A 2009, 27, 1126–1134. 10.1116/1.3186616.

[ref32] Park S.; Vohs J. M.; Gorte R. J. Direct oxidation of hydrocarbons in a solid-oxide fuel cel. Nature 2000, 404, 265–267. 10.1038/35005040. 10749204

[ref33] Graves C.; Ebbesen S. D.; Jensen S. H.; Simonsen S. B.; Mogensen M. B. Eliminating degradation in solid oxide electrochemical cells by reversible operation. Nat. Mater. 2015, 14, 239–244. 10.1038/nmat4165. 25532070

[ref34] Lintz H.-G.; Vayenas C. G. Solid ion conductors in heterogeneous catalysis. Angew. Chem., Int. Ed. 1989, 28, 708–715. 10.1002/anie.198907081.

[ref35] Stambouli A. B.; Traversa E. Solid oxide fuel cells (SOFCS): A review of an environmentally clean and efficient source of energy. Renewable Sustainable Energy Rev. 2002, 6, 433–455. 10.1016/S1364-0321(02)00014-X.

[ref36] Iannaci A.; Sciarria T. P.; Mecheri B.; Adani F.; Licoccia S.; D’Epifanio A. Power generation using a low-cost sulfated zirconium oxide based cathode in single chamber microbial fuel cells. J. Alloys Compd. 2017, 693, 170–176. 10.1016/j.jallcom.2016.09.159.

[ref37] Sayama K.; Arakawa H. Photocatalytic decomposition of water and photocatalytic reduction of carbon dioxide over zirconia catalyst. J. Phys. Chem. A 1993, 97, 531–533. 10.1021/j100105a001.

[ref38] Jacobson A. J. Materials for solid oxide fuel cells. Chem. Mater. 2010, 22, 660–674. 10.1021/cm902640j.

[ref39] Huang H. C.; Su P. C.; Kwak S. K.; Pornprasertsuk R.; Yoon Y. J. Molecular dynamics simulation of oxygen ion diffusion in yttria stabilized zirconia single crystals and bicrystals. Fuel Cells 2014, 14, 574–580. 10.1002/fuce.201300227.

[ref40] Park N.-G. Research Direction toward Scalable, Stable, and High Efficiency Perovskite Solar Cells. Adv. Energy Mater. 2020, 10, 1903106 10.1002/aenm.201903106.

[ref41] Lee M. M.; Teuscher J.; Miyasaka T.; Murakami T. N.; Snaith H. J. Efficient Hybrid Solar Cells Based on Meso-Superstructured Organometal Halide Perovskites. Science 2012, 338, 643–647. 10.1126/science.1228604. 23042296

[ref42] Hwang S. H.; Roh J.; Lee J.; Ryu J.; Yun J.; Jang J. Size-controlled SiO_2_ nanoparticles as scaffold layers in thin-film perovskite solar cells. J. Mater. Chem. A 2014, 2, 16429–16433. 10.1039/C4TA03087G.

[ref43] Bi D.; Moon S. J.; Häggman L.; Boschloo G.; Yang L.; Johansson E. M.; Nazeeruddin M. K.; Grätzel M.; Hagfeldt A. Using a two-step deposition technique to prepare perovskite (C_H_3N_H_3Pb_I_3) for thin film solar cells based on Zr_O_2 and Ti_O_2 mesostructures. RSC Adv. 2013, 3, 18762–18766. 10.1039/c3ra43228a.

[ref44] Larina L. L.; Alexeeva O. V.; Almjasheva O. V.; Gusarov V. V.; Kozlov S. S.; Nikolskaia A. B.; Vildanova M. F.; Shevaleevskiy O. I. Very wide-bandgap nanostructured metal oxide materials for perovskite solar cells. Nanosyst.: Phys. Chem. Math. 2019, 10, 70–75. 10.17586/2220-8054-2019-10-1-70-75.

[ref45] Vildanova M. F.; Nikolskaia A. B.; Kozlov S. S.; Karyagina O. K.; Larina L. L.; Shevaleevskiy O. I.; Almyasheva O. V.; Gusarov V. V. Nanostructured ZrO2–Y2O3-Based System for Perovskite Solar Cells. Dokl. Phys. Chem. 2019, 484, 36–38. 10.1134/S0012501619020040.

[ref46] Vildanova M. F.; Nikolskaia A. B.; Kozlov S. S.; Shevaleevskiy O. I.; Almjasheva O. V.; Gusarov V. V. Group IV Oxides for Perovskite Solar Cells. Dokl. Phys. Chem. 2021, 496, 13–19. 10.1134/S0012501621020020.

[ref47] Witoon T.; Chalorngtham J.; Dumrongbunditkul P.; Chareonpanich M.; Limtrakul J. CO_2_ hydrogenation to methanol over Cu/ZrO_2_ catalysts: Effects of zirconia phases. Chem. Eng. J. 2016, 293, 327–336. 10.1016/j.cej.2016.02.069.

[ref48] Wu C.; Lin L.; Liu J.; Zhang J.; Zhang F.; Zhou T.; Rui N.; Yao S.; Deng Y.; Yang F.; Xu W.; Luo J.; Zhao Y.; Yan B.; Wen X.-D.; Rodriguez J. A.; Ma D. Inverse Zr_O_2/Cu as a highly efficient methanol synthesis catalyst from C_O_2 hydrogenation. Nat. Commun. 2020, 11, 5767 10.1038/s41467-020-19634-8. 33188189 PMC7666171

[ref49] Yadav G. D.; Nair J. J. Sulfated zirconia and its modified versions as promising catalysts for industrial processes. Microporous Mesoporous Mater. 1999, 33, 1–48. 10.1016/S1387-1811(99)00147-X.

[ref50] Omarov S. O.; Sladkovskiy D. A.; Martinson K. D.; Peurla M.; Aho A.; Murzin D. Y.; Popkov V. I. Influence of the initial state of ZrO_2_ on genesis, activity and stability of Ni/ZrO_2_ catalysts for steam reforming of glicerol. Appl. Catal., A 2021, 616, 118098 10.1016/j.apcata.2021.118098.

[ref51] Dini F. W.; Helmiyati H.; Krisnandi Y. K. Cellulose and TiO_2_–ZrO_2_ nanocomposite as a catalyst for glucose conversion to 5-EMF. Bull. Chem. React. Eng. Catal. 2021, 16, 320–330. 10.9767/bcrec.16.2.10320.320-330.

[ref52] Tsoukalou A.; Serykh A. I.; Willinger E.; Kierzkowska A.; Abdala P. M.; Fedorov A.; Müller C. R. Hydrogen dissociation sites on indium-based ZrO_2_-supported catalysts for hydrogenation of CO_2_ to methanol. Catal. Today 2022, 387, 38–46. 10.1016/j.cattod.2021.04.010.

[ref53] Tsoukalou A.; Bushkov N. S.; Docherty S. R.; Mance D. A.; Serykh I.; Abdala P. M.; Copéret C.; Fedorov A.; Müller C. R. Surface Intermediates in In-Based ZrO_2_-Supported Catalysts for Hydrogenation of CO_2_ to Methanol. J. Phys. Chem. C 2022, 126, 1793–1799. 10.1021/acs.jpcc.1c08814.

[ref54] Zhang X.; Wu X.; Shi J. Additive manufacturing of zirconia ceramics: a state-of-the-art review. J. Mater. Res. Technol. 2020, 9, 9029–9048. 10.1016/j.jmrt.2020.05.131.

[ref55] Clarke D. R.; Levi C. G. Materials design for the next generation thermal barrier coatings. Annu. Rev. Mater. Res. 2003, 33, 383–417. 10.1146/annurev.matsci.33.011403.113718.

[ref56] Fiorentini V.; Gulleri G. Theoretical Evaluation of Zirconia and Hafnia as Gate Oxides for Si Microelectronics. Phys. Rev. Lett. 2002, 89, 266101 10.1103/PhysRevLett.89.266101. 12484834

[ref57] Wilk G. D.; Wallace R. M.; Anthony J. M. High-κ gate dielectrics: Current status and materials properties considerations. J. Appl. Phys. 2001, 89, 5243–5275. 10.1063/1.1361065.

[ref58] Stoneham A. M.; Gavartin J. L.; Shluger A. L. The oxide gate dielectric: do we know all we should?. J. Phys.: Condens. Matter 2005, 17, S2027–S2049. 10.1088/0953-8984/17/21/001.

[ref59] Kingon A. I.; Maria J. P.; Streiffer S. K. Alternative dielectrics to silicon dioxide for memory and logic devices. Nature 2000, 406, 1032–1038. 10.1038/35023243. 10984062

[ref60] Jeon T. S.; White J. M.; Kwong D. L. Thermal stability of ultrathin ZrO_2_ films prepared by chemical vapor deposition on Si(100). Appl. Phys. Lett. 2001, 78, 368–370. 10.1063/1.1339994.

[ref61] Copel M.; Gribelyuk M.; Gusev E. Structure and stability of ultrathin zirconium oxide layers on Si(001). Appl. Phys. Lett. 2000, 76, 436–438. 10.1063/1.125779.

[ref62] Zheng W.; Bowen K. H.; Li J.; Dąbkowska I.; Gutowski M. Electronic structure differences in ZrO_2_ vs HfO_2_. J. Phys. Chem. A 2005, 109, 11521–11525. 10.1021/jp053593e. 16354043

[ref63] Kim J. B.; Weichman M. L.; Neumark D. M. Structural isomers of Ti_2_O_4_ and Zr_2_O_4_ anions identified by slow photoelectron velocity-map imaging spectroscopy. J. Am. Chem. Soc. 2014, 136, 7159–7168. 10.1021/ja502713v. 24794915

[ref64] Kim J. B.; Weichman M. L.; Neumark D. M. High-resolution anion photoelectron spectra of TiO_2_^–^, ZrO_2_^–^, and HfO_2_^–^ obtained by slow electron velocity-map imaging. Phys. Chem. Chem. Phys. 2013, 15, 20973–20981. 10.1039/c3cp54084g. 24213351

[ref65] Brugh D. J.; Suenram R. D.; Stevens W. J. Fourier transform microwave spectroscopy of jet-cooled ZrO_2_ produced by laser vaporization. J. Chem. Phys. 1999, 111, 3526–3535. 10.1063/1.479674.

[ref66] Chertihin G. V.; Andrews L. Reactions of laser ablated titanium, zirconium, and hafnium atoms with oxygen molecules in condensing argon. J. Phys. Chem. A 1995, 99, 6356–6366. 10.1021/j100017a015.

[ref67] Le A.; Steimle T. C.; Gupta V.; Rice C. A.; Maier J. P.; Lin S. H.; Lin C.-K. The visible spectrum of zirconium dioxide, ZrO_2_. J. Chem. Phys. 2011, 135, 104303 10.1063/1.3632053. 21932889

[ref68] Li S.; Dixon D. A. Molecular structures and energetics of the (ZrO_2_)_n_ and (HfO_2_)_n_ (n = 1–4) clusters and their anions. J. Phys. Chem. A 2010, 114, 2665–2683. 10.1021/jp910310j. 20128586

[ref69] Woodley S. M.; Hamad S.; Mejías J. A.; Catlow C. R. A. Properties of small TiO_2_, ZrO_2_ and HfO_2_ nanoparticles. J. Mater. Chem. 2006, 16, 1927–1933. 10.1039/B600662K.

[ref70] Kaupp M. On the relation between π bonding, electronegativity, and bond angles in high-valent transition metal complexes. Chem. - Eur. J. 1999, 5, 3631–3643. 10.1002/(sici)1521-3765(19991203)5:123.3.co;2-t.

[ref71] Mok D. K. W.; Chau F.-t.; Dyke J. M.; Lee E. P. F. A combined ab initio and Franck–Condon simulation study of the photodetachment spectrum of ZrO_2_^–^. Chem. Phys. Lett. 2008, 458, 11–14. 10.1016/j.cplett.2008.03.037. 19060972

[ref72] Chen S.; Yin Y.; Wang D.; Liu Y.; Wang X. Structures, growth modes and spectroscopic properties of small zirconia clusters. J. Cryst. Growth 2005, 282, 498–505. 10.1016/j.jcrysgro.2005.05.017.

[ref73] Gong Y.; Zhang Q.; Zhou M. Matrix Isolation Infrared Spectroscopic and Theoretical Study of Group IV Metal Oxide Clusters: _M_2_O_2 and _M_2_O_4. J. Phys. Chem. A 2007, 111, 3534–3539. 10.1021/jp0711388. 17439109

[ref74] von Helden G.; Kirilyuk A.; van Heijnsbergen D.; Sartakov B.; Duncan M. A.; Meijer G. Infrared spectroscopy of gas-phase zirconium oxide clusters. Chem. Phys. 2000, 262, 31–39. 10.1016/S0301-0104(00)00267-6.

[ref75] Liang Z.; Wang W.; Zhang M.; Wu F.; Chen J. F.; Xue C.; Zhao H. Structural, mechanical and thermodynamic properties of ZrO_2_ polymorphs by first-principles calculation. Phys. B: Condens. Matter 2017, 511, 10–19. 10.1016/j.physb.2017.01.025.

[ref76] Delarmelina M.; Quesne M. G.; Catlow C. R. A. Modelling the bulk properties of ambient pressure polymorphs of zirconia. Phys. Chem. Chem. Phys. 2020, 22, 6660–6676. 10.1039/D0CP00032A. 32159203

[ref77] Boysen H.; Frey F.; Vogt T. Neutron powder investigation of the tetragonal to monoclinic phase transformation in undoped zirconia. Acta Crystallogr. Sect. B Struct. Sci. 1991, 47, 881–886. 10.1107/S010876819100856X.

[ref78] Fan Z.; Wang Y.; Zhang Y.; Liu J. Influence of oxygen vacancy compensation on the structure, electronic and mechanical properties of yttrium stabilized tetragonal zirconia. Mater. Sci. Semicond. Process. 2021, 135, 106082 10.1016/j.mssp.2021.106082.

[ref79] Smirnov M.; Mirgorodsky A.; Guinebretière R. Phenomenological theory of lattice dynamics and polymorphism of ZrO_2_. Phys. Rev. B 2003, 68, 104106 10.1103/PhysRevB.68.104106.

[ref80] Murti C. F. K.; Maslakah U.; Endarko E.; Triwikantoro T. Structural, physical and mechanical properties of zirconia-polymorph/alumina composites. Mater. Chem. Phys. 2022, 285, 126102 10.1016/j.matchemphys.2022.126102.

[ref81] Horti N. C.; Kamatagi M. D.; Nataraj S. K.; Wari M. N.; Inamdar S. R. Structural and optical properties of zirconium oxide (ZrO_2_) nanoparticles: Effect of calcination temperature. Nano Express 2020, 1, 010022 10.1088/2632-959X/ab8684.

[ref82] Balakrishnan K.; Veerapandy V.; Nalini V.; Vajeeston P. Density functional theory analysis of novel ZrO_2_ polymorphs: Unveiling structural stability, electronic structure, vibrational and optical properties. Comput. Mater. Sci. 2025, 246, 113439 10.1016/j.commatsci.2024.113439.

[ref83] Møller C.; Plesset M. S. Note on an Approximation Treatment for Many-Electron Systems. Phys. Rev. 1934, 46, 618–622. 10.1103/PhysRev.46.618.

[ref84] Head-Gordon M.; Pople J. A.; Frisch M. J. MP2 energy evaluation by direct methods. Chem. Phys. Lett. 1988, 153, 503–506. 10.1016/0009-2614(88)85250-3.

[ref85] Frisch M. J.; Head-Gordon M.; Pople J. A. A direct MP2 gradient method. Chem. Phys. Lett. 1990, 166, 275–280. 10.1016/0009-2614(90)80029-D.

[ref86] Dunning T. H. Jr. Gaussian basis sets for use in correlated molecular calculations. I. The atoms boron through neon and hydrogen. J. Chem. Phys. 1989, 90, 1007–1023. 10.1063/1.456153.

[ref87] Kendall R. A.; Dunning T. H. Jr.; Harrison R. J. Electron affinities of the first-row atoms revisited. Systematic basis sets and wave functions. J. Chem. Phys. 1992, 96, 6796–6806. 10.1063/1.462569.

[ref88] Andrae D.; Häussermann U.; Dolg M.; Stoll H.; Preuss H. Energy-adjusted ab initio pseudopotentials for the 2nd and 3rd row transition-elements. Theor. Chim. Acta 1990, 77, 123–141. 10.1007/BF01114537.

[ref89] Martin J. M. L.; Sundermann A. J. Correlation consistent valence basis sets for use with the Stuttgart–Dresden–Bonn relativistic effective core potentials: The atoms Ga–Kr and In–Xe. Chem. Phys. 2001, 114, 3408–3420. 10.1063/1.1337864.

[ref90] Cížek J. Advances in Chemical Physics; Hariharan P. C., Ed.; Wiley Interscience: New York, 1969; Vol. 14, p 35.

[ref91] Bartlett R. J.; Purvis G. D. III Many-body perturbation theory, coupled-pair many-electron theory, and the importance of quadruple excitations for the correlation problem. Int. J. Quantum Chem. 1978, 14, 561–581. 10.1002/qua.560140504.

[ref92] Purvis G. D. III; Bartlett R. J. A full coupled-cluster singles and doubles model: The inclusion of disconnected triples. J. Chem. Phys. 1982, 76, 1910–1918. 10.1063/1.443164.

[ref93] Scuseria G. E.; Janssen C. L.; Schaefer H. F. III An efficient reformulation of the closed-shell coupled cluster single and double excitation (CCSD) equations. J. Chem. Phys. 1988, 89, 7382–7387. 10.1063/1.455269.

[ref94] Foster J. P.; Weinhold F. Natural hybrid orbitals. J. Am. Chem. Soc. 1980, 102, 7211–7218. 10.1021/ja00544a007.

[ref95] Reed A. E.; Weinhold F. Natural bond orbital analysis of near-Hartree–Fock water dimer. J. Chem. Phys. 1983, 78, 4066–4073. 10.1063/1.445134.

[ref96] Reed A. E.; Weinstock R. B.; Weinhold F. Natural population analysis. J. Chem. Phys. 1985, 83, 735–746. 10.1063/1.449486.

[ref97] Carpenter J. E.; Weinhold F. Analysis of the geometry of the hydroxymethyl radical by the “different hybrids for different spins” natural bond orbital procedure. J. Mol. Struct.: THEOCHEM 1988, 169, 41–62. 10.1016/0166-1280(88)80248-3.

[ref98] Reed A. E.; Curtiss L. A.; Weinhold F. Intermolecular interactions from a natural bond orbital, donor-acceptor viewpoint. Chem. Rev. 1988, 88, 899–926. 10.1021/cr00088a005.

[ref99] Glendening E. D.; Badenhoop J. K.; Reed A. E.; Carpenter J. E.; Bohmann J. A.; Morales C. M.; Karafiloglou P.; Landis C. R.; Weinhold F. NBO 7.0.; Theoretical Chemistry Institute, University of Wisconsin: Madison, WI, 2018.

[ref100] Frisch M. J.; Trucks G. W.; Schlegel H. B.; Scuseria G. E.; Robb M. A.; Cheeseman J. R.; Scalmani G.; Barone V.; Petersson G. A.; Nakatsuji H.; Li X.; Caricato M.; Marenich A. V.; Bloino J.; Janesko B. G.; Gomperts R.; Mennucci B.; Hratchian H. P.; Ortiz J. V.; Izmaylov A. F.; Sonnenberg J. L.; Williams-Young D.; Ding F.; Lipparini F.; Egidi F.; Goings J.; Peng B.; Petrone A.; Henderson T.; Ranasinghe D.; Zakrzewski V. G.; Gao J.; Rega N.; Zheng G.; Liang W.; Hada M.; Ehara M.; Toyota K.; Fukuda R.; Hasegawa J.; Ishida M.; Nakajima T.; Honda Y.; Kitao O.; Nakai H.; Vreven T.; Throssell K.; Montgomery J. A. Jr.; Peralta J. E.; Ogliaro F.; Bearpark M. J.; Heyd J. J.; Brothers E. N.; Kudin K. N.; Staroverov V. N.; Keith T. A.; Kobayashi R.; Normand J.; Raghavachari K.; Rendell A. P.; Burant J. C.; Iyengar S. S.; Tomasi J.; Cossi M.; Millam J. M.; Klene M.; Adamo C.; Cammi R.; Ochterski J. W.; Martin R. L.; Morokuma K.; Farkas O.; Foresman J. B.; Fox D. J. Gaussian 16, Revision C.01; Gaussian, Inc.: Wallingford CT, 2016.

[ref101] Murad E.; Hildenbrand D. L. Thermochemical properties of gaseous ZrO and ZrO_2_. J. Chem. Phys. 1975, 63, 1133–1139. 10.1063/1.431439.

[ref102] Foltin M.; Stueber G. J.; Bernstein E. R. Investigation of the structure, stability, and ionization dynamics of zirconium oxide clusters. J. Chem. Phys. 2001, 114, 8971–8989. 10.1063/1.1359177.

