# Supplementary material for: Reversed Stability of Zirconium Oxide Dimer Isomers Triggered by Electron Gain or Removal
Source: Inorg Chem. 2025 Apr 3;64(14):7224–38. doi: 10.1021/acs.inorgchem.5c00964 (PMC12001248; doi:10.1021/acs.inorgchem.5c00964)
Supplement: Supplementary file 1 — ic5c00964_si_001.pdf [file ic5c00964_si_001.pdf]

Supporting Information

Reversed Stability of Zirconium Oxide Dimer Isomers Triggered by Electron Gain or Removal

Dawid Falkowski[1,2], Jakub Brzeski[1,2], Alicja Mikolajczyk[1,2], Piotr Skurski[1,2,3,*]

[1] *Faculty of Chemistry, University of Gdańsk, Wita Stwosza 63, 80-308 Gdańsk, Poland*

[2] *QSAR Lab Ltd., Trzy Lipy 3, 80-172, Poland*

[3] *Department of Chemistry, University of Utah, Salt Lake City, Utah 84112, U.S.A.*

[*] corresponding author: piotr.skurski@ug.edu.pl

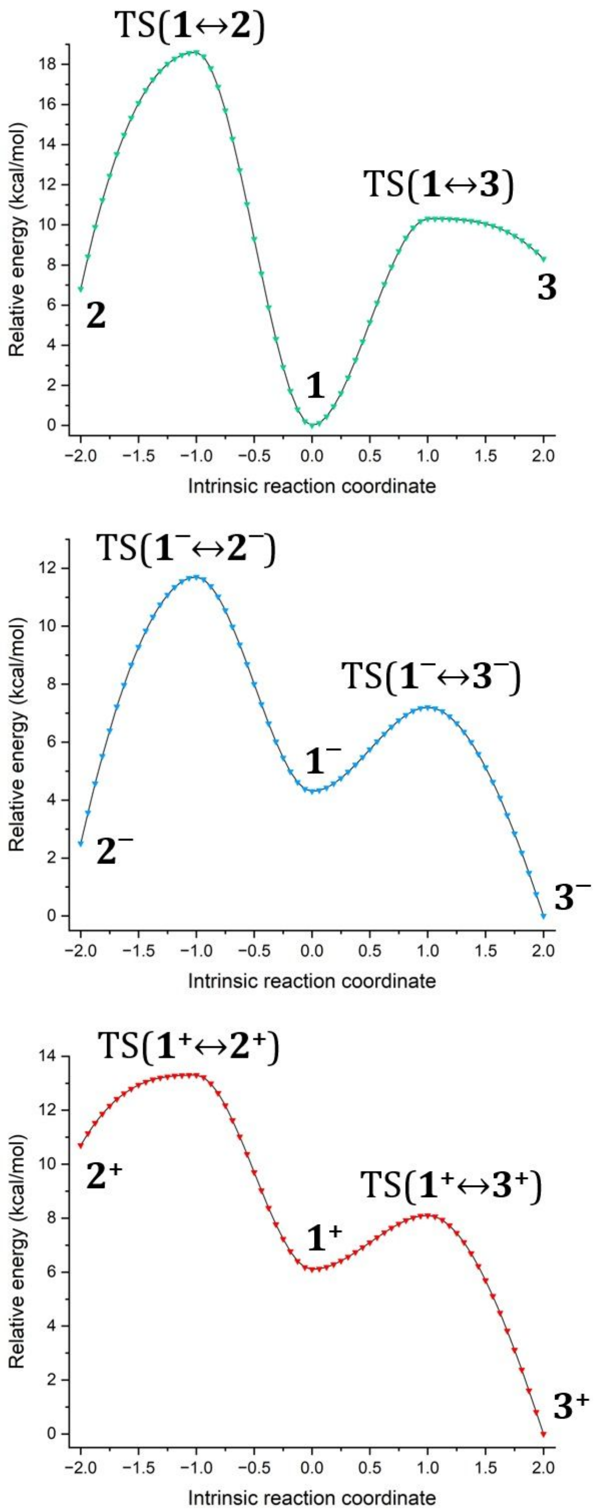

Figure S1. Relative energy profiles (in kcal/mol) along the intrinsic reaction coordinate (IRC) for the rearrangement pathways connecting the three isomeric forms of the zirconium oxide dimer: neutral (top), anionic (center), and cationic (bottom) species. Energies are given relative to the lowest-energy isomer in each case.

Table S1. Cartesian coordinates in Å of the isomeric structures of studied systems.

**1,** $(ZrO_2)_2$			
40	1.429347000	-0.360086000	-0.004022000
8	-0.000061000	-0.014577000	1.297411000
8	2.519968000	1.021207000	0.011445000
40	-1.429343000	0.360075000	0.004023000
8	-2.520011000	-1.021180000	-0.011445000
8	0.000081000	0.014604000	-1.297417000
**2,** $(ZrO_2)_2$			
40	-1.478276000	0.222151000	-0.000002000
8	-2.147448000	-1.401356000	0.000010000
8	-0.000009000	0.290613000	-1.301324000
40	1.478285000	0.222145000	-0.000004000
8	2.147405000	-1.401370000	0.000007000
8	0.000008000	0.290635000	1.301335000
**3,** $(ZrO_2)_2$			
40	0.000000000	0.000000000	-1.139315000
8	0.000000000	1.498243000	0.358575000
8	0.000000000	0.000000000	-2.906761000
40	0.000000000	0.000000000	1.505523000
8	-1.297516000	-0.749121000	0.358575000
8	1.297516000	-0.749121000	0.358575000
TS(**1**↔**2**), $(ZrO_2)_2$			
40	1.551554000	-0.000042000	0.253165000
8	0.076951000	1.326273000	0.209676000
8	2.229089000	0.000339000	-1.365419000
40	-1.399271000	-0.000035000	0.004965000
8	-3.144513000	0.000164000	-0.344132000
8	0.077059000	-1.326391000	0.209225000
TS(**1**↔**3**), $(ZrO_2)_2$			
40	1.141328000	0.049702000	-0.000210000
8	-0.303373000	-0.732404000	-1.293111000
8	2.901452000	-0.105718000	0.000451000
40	-1.505144000	-0.040259000	0.000152000
8	-0.303054000	-0.726665000	1.296253000
8	-0.475943000	1.517574000	-0.003300000
**1**$^-$, $(ZrO_2)_2^-$			
40	1.438769000	0.000008000	-0.348591000
8	-0.000034000	-1.310320000	0.000047000
8	2.677880000	-0.000043000	0.936420000
40	-1.438773000	-0.000005000	0.348581000
8	-2.677772000	-0.000017000	-0.936501000
8	-0.000055000	1.310364000	0.000083000
**2**$^-$, $(ZrO_2)_2^-$			
40	0.000000000	1.463054000	-0.218612000
8	0.000000000	2.600165000	1.155620000
8	-1.322103000	0.000000000	-0.062562000
40	0.000000000	-1.463054000	-0.218612000
8	0.000000000	-2.600165000	1.155620000
8	1.322103000	0.000000000	-0.062562000
**3**$^-$, $(ZrO_2)_2^-$			
8	0.000000000	0.000000000	-2.922140000
40	0.000000000	0.000000000	-1.116221000

40	0.000000000	0.000000000	1.510628000
8	0.000000000	1.508376000	0.316701000
8	1.306292000	-0.754188000	0.316701000
8	-1.306292000	-0.754188000	0.316701000
TS(**1**⁻↔**2**⁻), $(ZrO_2)_2^-$			
40	1.538650000	0.000004000	0.271360000
8	0.010828000	1.345644000	0.108436000
8	2.526691000	-0.000012000	-1.196671000
40	-1.405119000	-0.000003000	-0.030799000
8	-3.216010000	0.000003000	-0.223024000
8	0.010839000	-1.345641000	0.108451000
TS(**1**⁻↔**3**⁻), $(ZrO_2)_2^-$			
40	1.230972000	0.239915000	-0.000104000
8	-0.023141000	-0.487808000	-1.314829000
8	2.884914000	-0.444668000	0.000242000
40	-1.511694000	-0.261779000	0.000061000
8	-0.022901000	-0.487484000	1.314863000
8	-1.435262000	1.529276000	-0.000063000
**1**⁺, $(ZrO_2)_2^+$			
40	1.495498000	-0.353313000	0.049803000
8	0.000003000	-0.000007000	1.227388000
8	2.161105000	1.222481000	-0.157988000
40	-1.495486000	0.353331000	0.049787000
8	-2.161167000	-1.222486000	-0.157923000
8	-0.000005000	-0.000079000	-1.409427000
**2**⁺, $(ZrO_2)_2^+$			
40	-1.541884000	0.207331000	-0.017920000
8	-1.630763000	-1.510154000	-0.067928000
8	-0.000009000	0.596371000	-1.133244000
40	1.541894000	0.207332000	-0.017932000
8	1.630713000	-1.510211000	-0.067887000
8	0.000009000	0.350679000	1.448316000
**3**⁺, $(ZrO_2)_2^+$			
40	-1.094952000	0.000050000	0.000000000
8	0.285207000	0.689946000	-1.260602000
8	-2.992733000	-0.000202000	-0.000027000
40	1.522448000	-0.000095000	0.000011000
8	0.285085000	0.747149000	1.227564000
8	0.284956000	-1.436670000	0.033010000
TS(**1**⁺↔**2**⁺), $(ZrO_2)_2^+$			
40	1.591085000	0.000321000	0.231516000
8	-0.002351000	1.273123000	0.227204000
8	2.006203000	-0.003186000	-1.436174000
40	-1.356083000	0.000498000	0.051300000
8	-3.175908000	-0.002031000	-0.437199000
8	-0.002955000	-1.271998000	0.232092000
TS(**1**⁺↔**3**⁺), $(ZrO_2)_2^+$			
40	1.177727000	0.281415000	-0.028864000
8	-0.040183000	-0.523484000	-1.244644000
8	2.907415000	-0.650192000	0.051913000
40	-1.521646000	-0.261046000	0.018414000
8	0.051489000	-0.390721000	1.280977000
8	-1.199130000	1.462549000	-0.035996000
